# Fruit Peel Powder as Natural Antioxidant and Reinforcing Bio-Filler in Natural Rubber Latex Gloves: Cases of Mangosteen, Pomelo and Durian

**DOI:** 10.3390/antiox12051119

**Published:** 2023-05-18

**Authors:** Arkarapol Thumwong, Jitsuna Darachai, Nuatawan Thamrongsiripak, Shinji Tokonami, Tetsuo Ishikawa, Kiadtisak Saenboonruang

**Affiliations:** 1 Department of Materials Science, Faculty of Science, Kasetsart University, Bangkok 10900, Thailand; arkarapol.th@ku.th; 2 Special Research Unit of Radiation Technology for Advanced Materials (RTAM), Faculty of Science, Kasetsart University, Bangkok 10900, Thailand; jitsuna.d@ku.th; 3 Thailand Institute of Nuclear Technology (Public Organization), Nakhorn Nayok 26120, Thailand; nuatawan@tint.or.th; 4 Institute of Radiation Emergency Medicine, Hirosaki University, Aomori 0368564, Japan; tokonami@hirosaki-u.ac.jp; 5 Department of Radiation Physics and Chemistry, Fukushima Medical University, Fukushima 9601295, Japan; isikawat@fmu.ac.jp; 6 Department of Applied Radiation and Isotopes, Faculty of Science, Kasetsart University, Bangkok 10900, Thailand; 7 Kasetsart University Research and Development Institute, Kasetsart University, Bangkok 10900, Thailand; 8 Specialized Center of Rubber and Polymer Materials in Agriculture and Industry (RPM), Faculty of Science, Kasetsart University, Bangkok 10900, Thailand

**Keywords:** fruit peel powder, natural antioxidant, reinforcement, bio-fillers, natural rubber latex, latex gloves, waste, thermal aging, gamma irradiation

## Abstract

As the world is facing rapid increases in agricultural wastes that greatly affect global health, the environment, and economies, this work aims to alleviate such issues by introducing simple uses of waste fruit peel powder (FPP) derived from mangosteen (MPP), pomelo (PPP), or durian (DPP), as dual natural antioxidants and reinforcing bio-fillers in natural rubber latex (NRL) gloves. A thorough investigation was undertaken of the relevant characteristics for both FPP (morphological, functional groups, particle sizes, and thermals stability) and NRL gloves (morphological, functional groups, density, color, thermal stability, and mechanical properties—both before and after thermal/25 kGy gamma aging). The results indicated that the initial addition (2–4 parts per hundred parts of rubber by weight; phr) of FPP to NRL composites generally enhanced the strength and elongation at the break of the specimens, with the levels of the improvement varying depending on the type and content of FPPs. In addition to the reinforcing effects, the FPP also offered natural antioxidant properties, evidenced by higher values of aging coefficients for all FPP/NRL gloves under either thermal or 25 kGy gamma aging than those of pristine NRL. Furthermore, by comparing the tensile strength and elongation at break of the developed FPP/NRL gloves with the requirements for medical examination latex gloves according to ASTM D3578-05, the recommended FPP contents for actual glove production were 2–4 phr for MPP, 4 phr for PPP, and 2 phr for DPP. Consequently, based on the overall outcomes, the FPPs of interest showed promising potential for utilization as simultaneous natural antioxidants and reinforcing bio-fillers in NRL gloves, which would not only enhance the strength and ability of the gloves to resist oxidative degradation from heat and gamma irradiation but also increase their economical value as well as reducing the amounts of the investigated wastes.

## 1. Introduction

Rapid increases in the disposal of agricultural and food wastes have become global environmental and health concerns that are worsening the quality of living for both current and future generations [1,2]. To address these worrisome issues, various attempts have been made to reduce the amount of waste disposal, including the utilization of biodegradable materials and smart farming, as well as the recycling/upcycling of wastes for the production of advanced materials [3,4,5,6,7]. Among these wastes, fruit peels, which contain high contents of lignocellulosic biomass and bioactive compounds [8], can be reused in a variety of applications, including low-cost absorbent materials, edible coatings/films on food surfaces, natural antimicrobial agents, biological reduction agents for the synthesis of nanoparticles, and as an advanced organic resistive switching memory device [9,10,11,12,13,14].

In addition to these examples, fruit peels, which offer antioxidant properties due to their diverse compositions of phytochemicals, such as carotenoids and phenolic compounds, could replace some of the commercially available but highly toxic antioxidants used in conventional rubber products such as Wingstay L [15,16,17]. The importance of antioxidants is especially emphasized in the case of natural rubber (NR) products due to their highly unsaturated hydrocarbons of poly(*cis*-1,4-isoprene) in NR molecular chains that make them highly sensitive to oxidative degradation from ozone, heat, and radiation [18]. In particular, for natural latex gloves, the oxidative degradation during the process of product preparation, sterilization, and storage could noticeably reduce the usability and shelf-life of the gloves. For instance, the NRL films (without any antioxidants) suffered a great decrease in tensile strength from 13 MPa in a non-aged sample to less than 10 MPa in a thermal-aged sample (reduced by also 25%), which was lower than the requirement for latex gloves used in food and medical sectors [19]. This reduction in the mechanical properties was due to the lack of antioxidants in the samples that could convert free radicals into more stable products and end the degradation. Some examples of published studies reporting the promising antioxidant properties in fruit peels include the work of Suleria et al., who showed that the extracts from grapefruit peels, avocado peels, and mango peels had high radical-scavenging effects (RSEs) of 9.17 ± 0.19 mg (Ascorbic Acid Equivalent) AAE/g, 8.67 ± 0.44 mg AAE/g, and 8.67 ± 0.49 mg AAE/g, respectively, evaluated using a 2,2-diphenyl-1-picrylhydrazyl (DPPH) assay method [20]. In addition, Zhang et al. reported that the extracts from the peels of 19 different mandarins (*Citrus reticulata* Blanco) had an average RSE of 21.92 mg/g (dry weight) DW [21], implying a high antioxidant ability of the peels. Subsequently, based on the promising reports, Sukatta et al. explored the advantages of fruit peels as natural antioxidants by introducing extracted rambutan peel powder (eRPP) to a conventional vulcanized NR. The investigation indicated that the eRPP had similar antioxidant activities with those of a commercial antioxidant, namely *N*-(1,3-Dimethylbutyl)-*N*′-phenyl-*p*-phenylenediamine (6PPD), while the addition of at least 1 part per hundred parts of rubber by weight (phr) of eRPP to rubber vulcanizates resulted in the prepared rubber specimens having similar abilities to resist thermal aging and ozone aging as those using 6PPD and 2,2,4-trimethyl-1,2-dihydroquinoline (TMQ) at the same content [17].

Despite having high antioxidant properties, the procedure to introduce fruit peels to NR composites usually requires some hazardous chemicals and high heat to extract active compounds from the peels [17,22], consequently limiting the useability and processibility of the substances, as well as raising environmental and health concerns. To alleviate such drawbacks, a more environmentally friendly and simpler method to utilize fruit peels was introduced by Mahir and Ismail, who simply mixed 2 phr of dried mangosteen peel powder (MPP) with NR and then compared the mechanical properties of the NR specimens with those using commercial antioxidants. The results indicated that the thermal-aged MPP/NR composites had higher tensile strength and elongation at break than those of the NR with the commercial antioxidants, determined for the same filler content and aging conditions, implying the effectiveness of MPP as natural antioxidants [23]. Furthermore, their report offered a much simpler procedure to utilize fruit peels in NR products that reduced preparation steps and the amounts of chemicals/energy required in the common extraction procedure.

In addition to being natural antioxidants, fruit peels contain high contents of lignocellulosic biomass [24], making them suitable for the reinforcement of NR products. Generally, cellulosic fibers, such as those from oil palm, sisal, pineapple leaf, and banana stem, have been utilized as reinforcing bio-fillers in NR composites [25,26], as reported by Sivasubramanian et al., who showed that the addition of 35.1 wt% untreated pineapple leaf fibers (PALF) to NR composites enhanced tensile strength, tear strength, and hardness (Shore A) from ~10 MPa, ~32 N/mm, and ~35, respectively, in pristine NR to ~11 MPa, ~79 N/mm, and ~68, respectively, in the PALF/NR composites [27], indicating the reinforcing effects of adding cellulosic fibers to NR composites. However, in contrast to fibers, there are only a few available reports of fruit peels being utilized as reinforcing bio-fillers in NR products. For instance, Mahir and Ismail, who added 2 phr dried MPP to NR, reported that the tensile modulus at 100% elongation, tensile strength, and elongation at break was enhanced from 55.83 MPa, 6.04 MPa, and 27.30%, respectively, in pristine NR to 138.99 MPa, 26.54 MPa, and 44.64%, respectively, in the MPP/NR composites [23], clearly showing the reinforcing effects of MPP on NR products, which could widen potential applications of NRL products to those requiring high strength and elongation at break, especially in medical, electronic, and food sectors.

As mentioned above, the great potential and attractive benefits of fruit peels as natural antioxidants and reinforcing bio-fillers, as well as the limited availability of in-depth reports on their utilization in NR products, have driven the need for wider and more thorough investigations. Hence, the current work aimed to investigate the dual antioxidant and reinforcing effects of fruit peel powder (FPP), derived from mangosteen (*Garcinia mangostana*), pomelo (*Citrus maxima*), and durian (*Durio zibethinus* L.), on the properties of NRL gloves. The properties of interest for FPPs investigated in this work included morphological (scanning electron microscopy; SEM), active functional groups (Fourier-transform infrared spectroscopy; FTIR), particle sizes, and thermal stability (thermogravimetric analysis; TGA) properties, while the properties of interest for FPP/NRL gloves with varying FPP contents from 0 to 2, 4, and 6 phr included morphological (SEM), active functional groups (FTIR), physical (density and color index), thermal stability (TGA), and mechanical (tensile modulus, tensile strength, and elongation at break—both before and after thermal/25 kGy gamma aging) properties. It should be noted that the gamma aging of 25 kGy on the specimens was carried out in this work in order to replicate the common method to sterilize single-use NRL gloves via gamma irradiation. The outcomes from this work not only present valuable information on the antioxidant and reinforcing properties of the FPP on NRL gloves but also offer a simpler and more environmentally friendly method to utilize waste fruit peels in rubber products, which could be used as a basis for the development of other materials.

## 2. Materials and Methods

### 2.1. Materials and Chemicals

High-ammonia natural rubber latex (HA-NRL) was supplied by the Office of Rubber Authority of Thailand (RAOT), Bangkok, Thailand. The HA-NRL used for sample preparation had total solid and dry rubber contents of 61.0% (ISO 124:2014) and 60.3% (ISO 126:2005), respectively [28]. Other chemicals, with their respective contents and roles, are shown in Table 1. The fruit peel powder (FPP), consisting of mangosteen peel powder (MPP), pomelo peel powder (PPP), and durian peel powder (DPP), was obtained from local fruit markets in Bangkok, Thailand; distilled water was supplied by the Faculty of Science, Kasetsart University, Bangkok, Thailand; and other chemicals were supplied by the RAOT (Bangkok, Thailand). Prior to the sample preparation, all chemicals and substances except KOH and Teric 16A16 were prepared into solutions by mixing each chemical with ammonia, vultamol, bentonite, and distilled water for 24 h in a stainless-steel ball mill (the final weight content for FPP-to-ammonia-to-vultamol-to-bentonite-to-distilled water was 10:10:1:1:78, while the final weight content for other chemical-to-vultamol-to-bentonite-to-distilled water was 50:1:1:48). This additional step in preparing chemical solutions was introduced to ensure improved compatibility between all chemicals and the NRL matrix during the sample preparation, which could reduce voids and particle agglomerations as well as enable the chemicals to perform at their fullest capacities. Notably, the formulation and procedure for sample preparation in this work were based on our previous work relating to the development of X-ray shielding NRL gloves [16,28], while FPPs were introduced to the formulation in order to determine their potential to replace conventional antioxidants in NR products such as 6PPD and TMQ [17].

**Figure 1:**
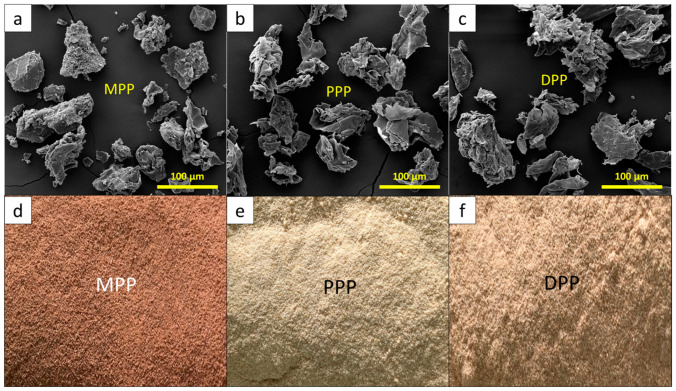
Images of (**a**)/(**d**) MPP, (**b**)/(**e**) PPP, and (**c**)/(**f**) DPP. (**a**–**c**) are micrograph images taken using SEM with 400× magnification, while (**d**–**f**) are optical images showing actual colors of ground MPP, PPP, and DPP.

To prepare FPP for sample preparation, the obtained mangosteen peels, pomelo peels, and durian peels were cut into small pieces and dried under sunlight for 2–3 days. Then, the dried fruit peels were ground into fine particles using a grinder (HR-1500W, Energy789 Co., Ltd.; Bangkok, Thailand) and passed through a 60-mesh sieve. Micrograph images using scanning electron microscopy (SEM; Quanta 450 FEI JSM-6610LV; Eindhoven, The Netherlands) as well as optical images of the prepared MPP, PPP, and DPP are shown in Figure 1, which indicates that the FPPs were irregular flakes, with average ± standard deviation particle sizes of 71.9 ± 8.8 µm, 62.2 ± 8.0 µm, and 83.4 ± 5.5 µm for MPP, PPP, and DPP, respectively (determined using the ImageJ software version 1.50i, provided by the National Institutes of Health and the Laboratory for Optical and Computational Instrumentation, University of Wisconsin, Madison, WI, USA). Figure 1d–f shows that the colors of ground MPP, PPP, and DPP were roughly maroon, off-white, and light brown, respectively.

### 2.2. Preparation of FPP/NRL Mixture

The NRL was continuously stirred using an automatic top stirrer (Eurostar 60 digital; IKA; Bangkok, Thailand) at a rotation speed of 300 rpm for 60 min. Next, KOH, Teric 16A16, S, ZDEC, ZMBT, ZnO, and distilled water were added sequentially to NRL with a 2 min interval between each chemical, after which the mixture was continuously stirred for another 60 min. Then, the NRL mixture was stored in a closed container at room temperature for 72 h to allow the pre-vulcanization process to occur, prior to the addition of each FPP into separate mixtures. After the addition of FPP, the stirring was continued for another 60 min, and the mixture was later kept in a closed container for further steps.

### 2.3. Preparation of FPP/NRL Gloves

To prepare the FPP/NRL gloves, the ceramic molds were thoroughly washed, rinsed, and oven-dried at 80 °C for 40 min. Then, the molds were dipped in a 35% coagulant, consisting of Ca(NO_3_)_2_, Teric 16A16, 50% CaCO_3_, and distilled water (RAOT, Bangkok, Thailand) with final weight contents of 35.0:0.1:5.0:59.9, respectively, for 5 s to improve rubber adhesion to the molds [16,28,29]. After coagulant dipping, the molds were oven-dried at 80 °C for 2 min before being dipped in the FPP/NRL mixture for 30 s, carefully flicked, rotated (multiple times), and oven-dried at 110 °C for 50 min. Lastly, the FPP/NRL gloves were peeled off the molds and kept in polyethylene (PE) bags with a relative humidity of 55–60% and a temperature of 24–26 °C for further use and testing.

### 2.4. Thermal and Gamma Aging of FPP/NRL Gloves

To determine the ability of the FPP/NRL gloves to resist oxidative degradation due to heat, the samples were placed in a hot-air oven at 100 °C for 22 h, following the ASTM D3578-19 standard testing procedure [30]. In addition, since latex gloves used in medical, electronic, and food applications are usually required to be sterilized to prevent the growth of microorganisms on the glove surface [31], the effects of gamma aging on the FPP/NRL gloves were investigated by irradiating all samples with 25 kGy gamma rays using a ^60^Co source at the Thailand Institute of Nuclear Technology (Public Organization) (Nakhon Nayok, Thailand). The changes in mechanical properties for all samples under thermal and gamma aging were determined and compared with non-aged samples. Notably, the dose of 25 kGy was selected for gamma aging because typical doses for sterilization of medical devices are in the range of 25–50 kGy [32].

### 2.5. Characterization

#### 2.5.1. Thermal Stability and Functional Groups

To determine the thermal stability of both the FPPs and FPP/NRL gloves, thermogravimetric analysis (TGA; TGA/DSC 3+; Mettler-Toledo; Greifensee, Switzerland) was conducted on all samples using a temperature range of 30–700 °C. The procedure was conducted at a heating rate of 10 °C/min under a nitrogen atmosphere [33]. The active functional groups of the FPPs and FPP/NRL gloves were determined using Fourier-transform infrared spectroscopy (FTIR) (Vertex 70, Bruker, San Jose, CA, USA), with wavenumbers in the range of 500–4000 cm^−1^.

#### 2.5.2. Morphology, Density, and Color

The morphology of the FPP/NRL gloves was determined using SEM (Quanta 450 FEI, JSM-6610LV; Eindhoven, The Netherlands). All samples were coated with gold to improve the signal-to-noise ratio using a sputter coater (Quorum SC7620; Mini Sputter Coater/Glow Discharge System; Laughton, UK) prior to taking the SEM images [34].

The density of the FPP/NRL gloves was determined using a densitometer (MH-300A; Shanghai, China) with a precision of 0.001 g/cm^3^. The determination was carried out based on Archimedes’ principle [35].

The colors of the FPP/NRL gloves were systematically determined using a color index measurement based on UV-Vis-NIR spectrometry (SolidSpec-3700; Shimadzu; Kyoto, Japan) in transmittance mode with a wavelength range of 380–750 nm and a standard lighting source *C*, following the CIE-L*a*b* colorimetric method recommended by the Commission Internationale de l’Eclairage (CIE) (Vienna, Austria). The color parameters used in this work were L*, a*, and b*, which represent the color positions of the samples in a lightness axis, a blue–yellow axis, and a green–red axis, respectively [36]. To compare changes in colors after the addition of each FPP to the NRL gloves, the color difference (ΔE) between a pristine NRL glove and each FPP/NRL glove was calculated using Equation (1):

(1) $$∆E=\sqrt{{\left(L_{a}^{*}-L_{o}^{*}\right)}^{2}+{\left(a_{a}^{*}-a_{o}^{*}\right)}^{2}+{\left(b_{a}^{*}-b_{o}^{*}\right)}^{2}}$$

where the parameters with the subscripts “_a_” and “_o_” represent the values obtained from aged samples and non-aged samples, respectively. It should be noted that the measurements of density and color for each NRL glove were carried out with at least three repetitions.

#### 2.5.3. Radical-Scavenging Capacity

The antioxidant properties of the FPPs were determined based on their radical-scavenging capacities using a DPPH assay method [37]. To carry out the measurement, each FPP was extracted using 99.9% ethanol [38], for which the volumes of ethanol were selected such that the final concentrations of:

- MPP extracts were 0.0125, 0.025, 0.05, or 0.1 mg/mL;
- PPP extracts were 0.156, 0.625, 2.5, or 10 mg/mL;
- DPP extracts were 0.781, 3.125, 12.5, or 50 mg/mL.

Then, 0.1 mM of DPPH was allowed to react with the same volume of FPP extracts (1:1, *v*/*v*) under continuous stirring in a dark chamber for 30 min. Lastly, the absorbance at 517 nm was measured using a UV-Vis spectrometer (Cary 100 Scan; Thai Unique Co., Ltd.; Bangkok, Thailand) for each extract, and the antioxidant properties of the FPP were then determined based on the radical-scavenging effect (RSE), calculated using Equation (2):

(2) $$RSE=\left(\frac{A_{c}-A_{s}}{A_{c}}\right)\times 100\%$$

where A_c_ and A_s_ are the absorbances for a control (only DPPH) and an FPP extract, respectively [39]. The measurement of RSE was carried out for at least three repetitions for each sample.

#### 2.5.4. Mechanical Properties

The mechanical properties, consisting of tensile modulus at 500% elongation (TM500), tensile strength (TS), and elongation at break (EB), of non-aged and aged FPP/NRL gloves were determined using a universal testing machine (TM-G5K; TM Tech; Bangkok, Thailand) according to ASTM D412-06 standard testing procedures. The tensile testing speed used for the measurement was 500 mm/min, and each sample was cut into a dumbbell shape (die C) prior to the measurement.

To determine the ability to resist oxidative degradation from heat and radiation based on levels of change in the mechanical properties of the samples after aging, the aging coefficient (C_aging_) for each sample formulation and aging condition were determined using Equation (3):

(3) $$C_{aging}=\frac{{TS}_{a}\times {EB}_{a}}{{TS}_{o}\times {EB}_{o}}$$

where the parameters with the subscripts “_a_” and “_o_” represent the values obtained from aged samples and non-aged samples, respectively [40]. It should be noted that the measurement of mechanical properties for both non-aged and aged samples was carried out with at least three repetitions.

### 2.6. Statistical Analysis

A level of 95% significance (*p* < 0.05) was used for the descriptive analysis of the data. The *t*-test was also applied to determine any significant difference between the results of interest. The statistical analysis was conducted using the statistical analysis package available in Microsoft Excel (New York, NY, USA), and the results are shown in the Supplementary Materials (Tables S1–S26).

## 3. Results and Discussion

### 3.1. Characteristics of FPP

#### 3.1.1. Thermal Stability

**Figure 2:**
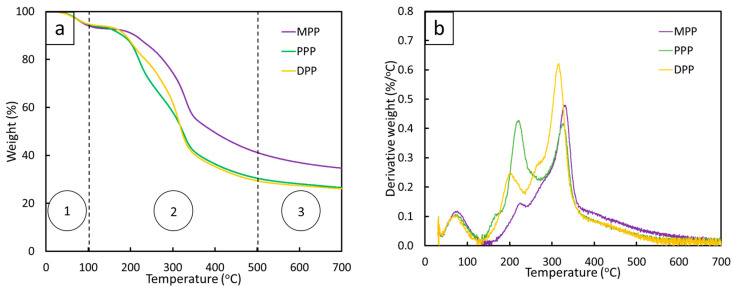
Thermal stabilities of FFPs (MPP, PPP, and DPP) determined using thermogravimetric analysis (TGA) with (**a**) correlations between remaining weights of each FPP and temperature, where circled numbers indicate stages of thermal stabilities for FPPs and (**b**) correlations between derivative weights of FPPs and temperature.

The thermal stabilities of MPP, PPP, and DPP were determined based on TGA. The results, as shown in Figure 2, indicated that the thermal stabilities of MPP, PPP, and DPP could be divided into three stages. In the first stage, which occurred at temperatures lower than 100 °C (~80 °C), the initial weight losses were due to the release of weak-bonded water molecules in the amorphous region of the FPP [41,42]. The second stage, with the peaks of derivative weights at 200–220 °C, 315–330 °C, and 400–420 °C (Figure 2b), was related to the decompositions of hemicellulose, cellulose, and lignin, respectively, in the FPP [41,43]. In the third stage, the weights of all FPPs were relatively unchanged above 500 °C, corresponding to the remains of residue from biochar, mostly composed of lignin [41]. Notably, MPP had a greater amount of remaining residue (35%) than PPP (27%) and DPP (26%). This could have been due to MPP containing higher contents of lignin than PPP and DPP, resulting in more remaining biochar after the decomposition (contents of lignin in MPP, PPP, and DPP were reported to be 48.52%, 3.16%, and 13.6%, respectively [44,45,46]).

#### 3.1.2. Functional Groups

**Figure 3:**
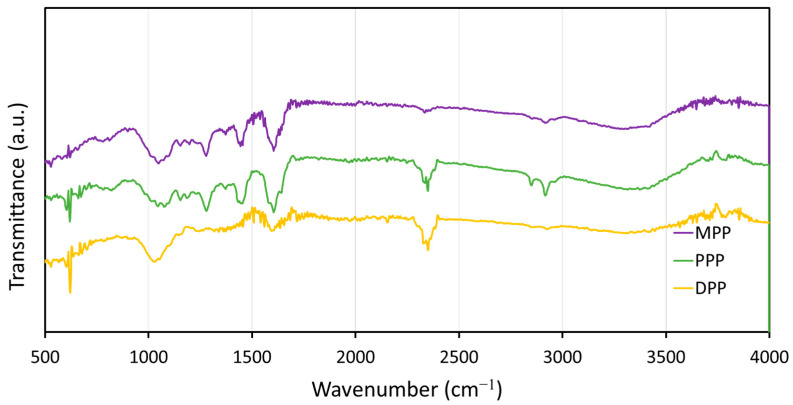
Functional groups of the FPPs (MPP, PPP, and DPP), determined using Fourier-transform infrared spectroscopy (FTIR).

The functional groups of all FPPs, including MPP, PPP, and DPP, determined using FTIR, are shown in Figure 3. The results indicated that all three FPPs exhibited similar active functional groups (only slight differences in magnitudes), with the dominant and important peaks at 1050, 1280, 1446, 1608, 2850, 2920, and 3330 cm^−1^. The first four peaks at 1050, 1280, 1446, and 1608 cm^−1^ corresponded to the vibrations of C–O from primary alcohols and those of C–O, C–H, and C=O from ketone, respectively. The next two peaks at 2850 and 2920 cm^−1^ were due to the vibrations of C–H from methylene groups and methyl groups. The last two dominant peaks at 2920 and 3330 cm^−1^ were related to the vibrations of O–H from hydroxyl groups, commonly found in cellulose, lignin, and pectin in the FPPs [47].

#### 3.1.3. Radical-Scavenging Capacity

**Figure 4:**
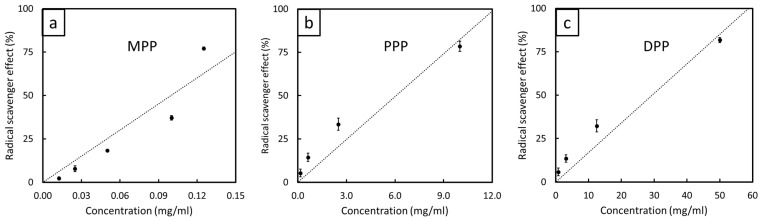
Radical-scavenging effects (RSEs) of extracts from (**a**) MPP, (**b**) PPP, and (**c**) DPP with varying extract concentrations. The error bars represent standard deviations of the mean.

The RSEs of MPP, PPP, and DPP, determined using a DPPH assay method, are shown in Figure 4, which indicates a strong, positive, linear correlation between the RSE and FPP concentration, with R^2^ values for MPP, PPP, and DPP being 0.940, 0.962, and 0.972, respectively. To estimate and compare the antioxidant capacities of all FPPs in this work, the values of IC_50_ (the concentration required to obtain 50% of the initial RSE) were determined from the fitted correlations in Figure 4, with the calculated values shown in Table 2. The results revealed that MPP (DPP) had the lowest (highest) IC_50_ value of 0.1 mg/mL (29.41 mg/mL) (95% confidence level—Table S1), implying the highest (lowest) antioxidant capacity in MPP (DPP). Since bioactive compounds, such as polyphenols (flavonoids, phenolic acid, and lignans), play important roles in defining the antioxidant activity of substances by reacting with free radicals and converting them into more stable products [38], it was reasonable to assume (based on the IC_50_ values) that MPP contained higher total polyphenolic compounds than PPP or DPP. This was consistent with the works of Wittenauer et al., Xiao et al., and Zhan et al., who reported that MPP, PPP, and DPP contained total polyphenolic compounds of 65.36, 17.99–53.64, and 1.86 mg/g DW, respectively [48,49,50], which made MPP a better antioxidant than PPP and DPP. It should be noted that the equations obtained from the correlations between the RSE and FPP concentration in Figure 4 are shown in Equations (4)–(6):

(4) $${RSE}_{MPP}=501.21\times conc.$$

(5) $${RSE}_{PPP}=8.23\times conc.$$

(6) $${RSE}_{DPP}=1.70\times conc.$$

where RSE_MPP_, RSE_PPP_, RSE_DPP_, and conc. are the radical-scavenging effects of MPP (%), the radical-scavenging effects of PPP (%), the radical-scavenging effects of DPP (%), and the corresponding extract concentrations (mg/mL), respectively. These obtained equations were then used for the calculation of IC_50_ for each type of FPP.

### 3.2. Characteristics of FPP/NRL Gloves

#### 3.2.1. Colors

**Figure 5:**
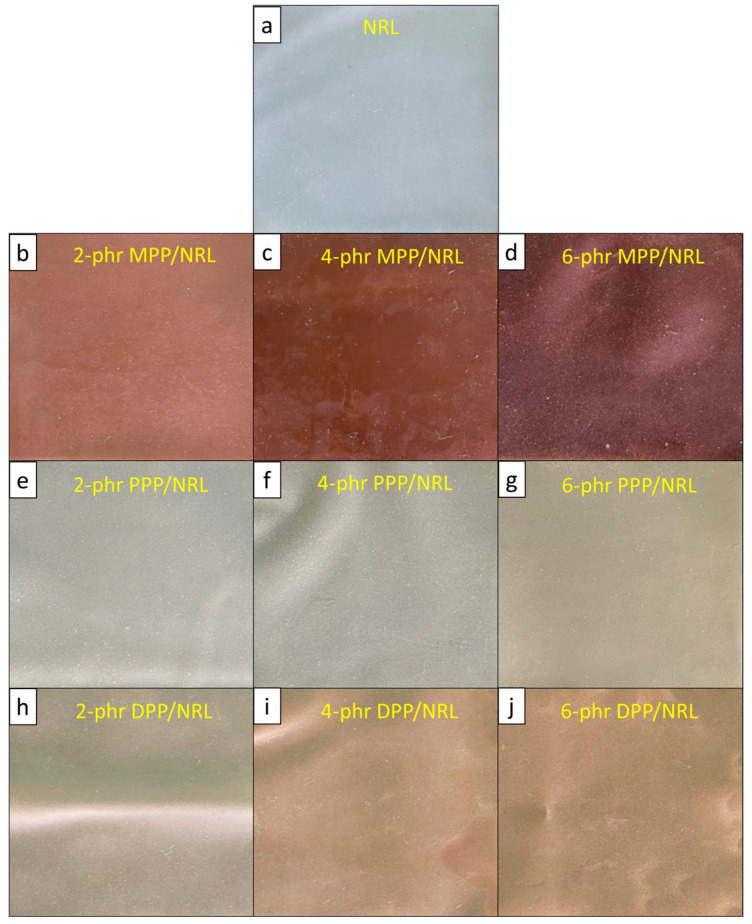
Optical images of (**a**) NRL, (**b**) 2 phr MPP/NRL, (**c**) 4 phr MPP/NRL, (**d**) 6 phr MPP/NRL, (**e**) 2 phr PPP/NRL, (**f**) 4 phr PPP/NRL, (**g**) 6 phr PPP/NRL, (**h**) 2 phr DPP/NRL, (**i**) 4 phr DPP/NRL, and (**j**) 6 phr DPP/NRL gloves.

For the MPP/NRL, PPP/NRL, and DPP/NRL gloves, the surface colors and color indices (determined using UV-Vis-NIR spectrometry) are shown in Figure 5 and Table 3, respectively. As shown in Figure 5, the colors of samples clearly changed after the addition of the FPPs to the NRL, with the levels of color change (ΔE) increasing at higher FPP contents (95% confidence level—Tables S2–S4) [51]. Specifically, for the MPP/NRL gloves, which had the most pronounced color changes after the addition of MPP (ΔE as high as 53.1 in the sample containing 6 phr MPP), the values of L* (a* and b*) noticeably decreased (increased) with increasing MPP contents, indicating that the samples became darker (redder and yellower, respectively), which agreed with the actual color of the ground MPP shown in Figure 1d.

#### 3.2.2. Densities

The densities of all FPP/NRL glove samples, shown in Table 4, increased with the addition of FPPs to the glove (95% confidence level—Table S5) due to the higher densities of lignin and cellulose in the FPP (densities of lignin and cellulose have been reported to be 1.35–1.50 g/cm^3^ and 1.58–1.60 g/cm^3^, respectively [52]) than those of NRL (0.90 ± 0.02 g/cm^3^), resulting in an increase in the sample mass for a given volume, after the addition of FPP to the NRL composites.

#### 3.2.3. Thermal Stability

**Figure 6:**
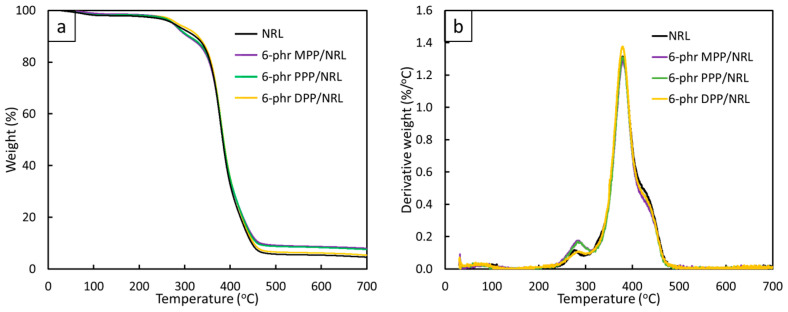
Thermal stability of pristine NRL and NRL composites containing 6 phr of FPP, determined using TGA, with (**a**) correlations between remaining weight of NRL composites and temperature, and (**b**) correlations between derivative weight of NRL composites and temperature.

The thermal stabilities of pristine NRL, 6 phr MPP/NRL, 6 phr PPP/NRL, and 6 phr DPP/NRL composites, determined using TGA, are shown in Figure 6. The results revealed that all samples had similar weight loss and thermal stability (Figure 6a), implying that the addition of FPP did not noticeably alter thermal stability in the NRL composites, with the peaks of the derivative weights (Figure 6b) being 80 °C, 280–290 °C, 380, and 400–420 °C. The first peaks at 80 °C corresponded to the release of water molecules that were weakly bound to the NRL matrix and FPP, while the peaks at 280–290 °C and 400–420 °C corresponded to the decompositions of hemicellulose, cellulose, and lignin from the added FPP. Lastly, the peaks at 380 °C, which had the highest magnitude of derivative weight loss, corresponded to the decomposition of pure NRL (the main matrix) [53]. Notably, the remaining biochar in 6 phr MPP/NRL (8.2%) was higher than that in the pristine NRL (4.5%), 6 phr PPP/NRL (7.5%), and 6 phr DPP/NRL (4.8%), probably due to MPP having the highest remaining residue in comparison to PPP and DPP, as shown in Figure 2a.

#### 3.2.4. Functional Groups

**Figure 7:**
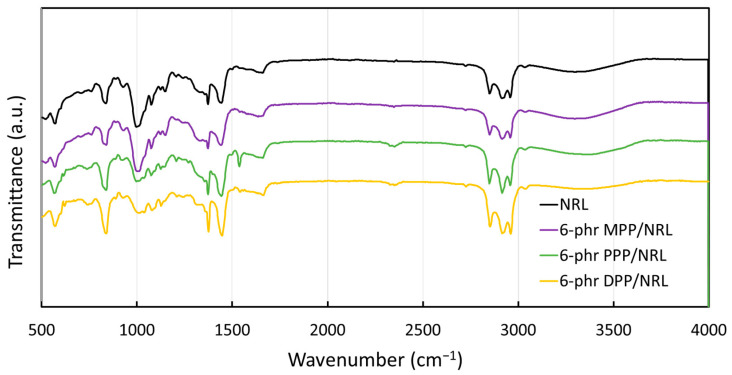
Functional groups of the pristine NRL and NRL containing 6 phr of MPP, PPP, and DPP determined using Fourier-transform infrared spectroscopy (FTIR).

The functional groups (determined using FTIR) of pristine NRL and NRL gloves containing 6 phr of MPP, PPP, and DPP are shown in Figure 7. The results indicated that all samples had similar dominant peaks (with slight differences in magnitudes), with the important peaks being at 841, 1013, 1080, 1128, 1153, 1375, 1446, 1664, 2850, 2932, 2964, and 3350 cm^−1^. The peaks at 841, 1375, 2932, and 2964 cm^−1^ corresponded to the vibrations of C–H from methyl groups (CH_3_), while the peaks at 1446 and 2850 cm^−1^ corresponded to the vibrations of C–H from methylene groups (CH_2_), for which these two groups are parts of the NR molecular chains ((C_5_H_8_)_n_). In addition, the dominant peaks at 1013, 1080, 1128, and 1153 cm^−1^ were due to the vibrations of C–C, and the peak at 1664 cm^−1^ was due to the vibration of C=C in the NR molecular backbones. The last peak at 3350 cm^−1^ corresponded to the vibration of O–H from water molecules as well as lignocellulose in the samples [54]. It should be noted that the addition of FPPs to NRL did not substantially alter active functional groups in this work, probably due to only small FPP contents (up to 6 phr) being added to the NRL matrix.

#### 3.2.5. Mechanical Properties

**Figure 8:**
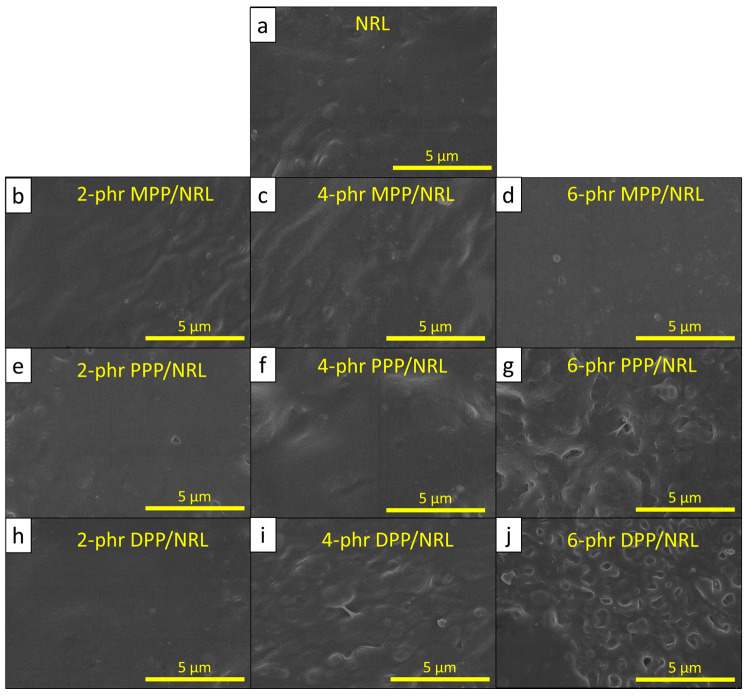
Micrograph images showing morphological properties of (**a**) NRL, (**b**) 2 phr MPP/NRL, (**c**) 4 phr MPP/NRL, (**d**) 6 phr MPP/NRL, (**e**) 2 phr PPP/NRL, (**f**) 4 phr PPP/NRL, (**g**) 6 phr PPP/NRL, (**h**) 2 phr DPP/NRL, (**i**) 4 phr DPP/NRL, and (**j**) 6 phr DPP/NRL gloves (10,000× magnification).

The mechanical properties, consisting of a tensile modulus at 500% elongation (TM500), tensile strength (TS), and elongation at break (EB), of the NRL composites containing varying FPP contents of 0, 2, 4, or 6 phr are shown in Table 5. The results indicated that TM500 generally increased with increasing MPP and DPP contents (mainly due to the higher rigidity of MPP and DPP than that of NR), subsequently enhancing overall rigidity and the TM500 value of the NRL composites. However, in contrast to the MPP/NRL and DPP/NRL samples, the TM500 value of the PPP/NRL gloves initially increased with the initial addition of 2 phr due to the rigidity of PPP (similar to MPP and DPP) but later decreased at higher PPP contents (95% confidence level). This reduction in TM500 could have been due to the release of gases, such as CO, H_2_, and C_x_H_y_, during the oven-drying that created large air bubbles and visible voids in the samples (Figure 8g) [8], subsequently reducing the overall rigidity and the TM500 value of the PPP/NRL composites.

Table 5 also shows the reinforcing effects of FPP in NRL composites, as evidenced by the increases in TS of the NRL gloves containing 4 phr MPP, 4 phr PPP, and 2 phr DPP (95% confidence level—Table S6). The initial reinforcing effects of FPP found in this work were in good agreement with other studies [17,55]. However, as more FPP was added to the NRL composites, the TS began to decrease. This negative effect from adding too much FPP could have been due to the initiation of particle agglomerations due to the unfavorable filler–filler interactions, as well as the creation of air bubbles and voids that reduced the matrix continuity and the ability to transfer and withstand external forces, thus affecting the strength of the composites [56]. In addition to TM500 and TS, the EB of the NRL composites mostly decreased with increasing FPP contents (except those containing PPP), which could have been due to the obstruction from the added FPP that limited the abilities of NR chains to mobilize along the directions of external forces [57].

**Figure 9:**
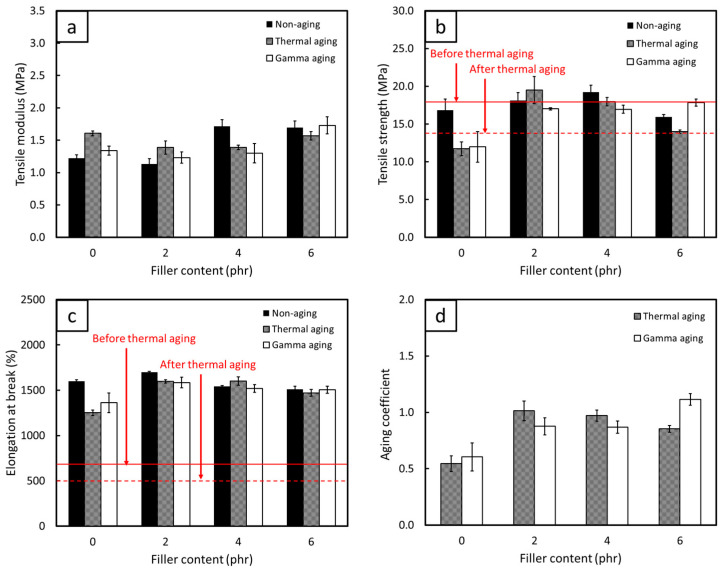
Mechanical properties of (**a**) tensile modulus at 500% elongation, (**b**) tensile strength, (**c**) elongation at break, and (**d**) aging coefficient of thermal-aged and gamma-aged NRL gloves containing varying contents of MPP. The solid and dotted red lines in (**b**,**c**) represent minimum requirements for non-aged and thermal-aged medical examination latex gloves, respectively, according to ASTM D3578-05. The error bars represent standard deviations of the mean values.

**Figure 10:**
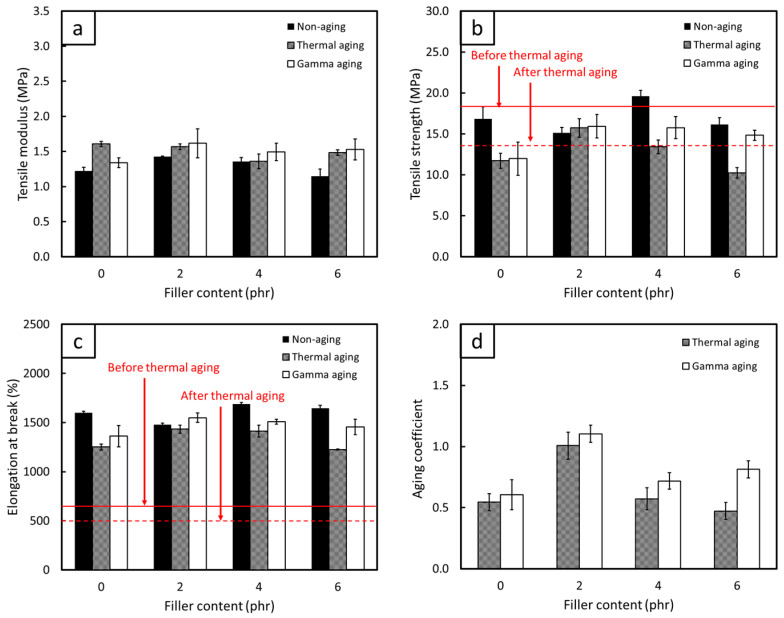
Mechanical properties of (**a**) tensile modulus at 500% elongation, (**b**) tensile strength, (**c**) elongation at break, and (**d**) aging coefficient of non-aged, thermal-aged, and gamma-aged NRL gloves containing varying contents of PPP. The solid and dotted red lines in (**b**,**c**) represent minimum requirements for non-aged and thermal-aged medical examination latex gloves, respectively, according to ASTM D3578-05. The error bars represent standard deviations of mean values.

**Figure 11:**
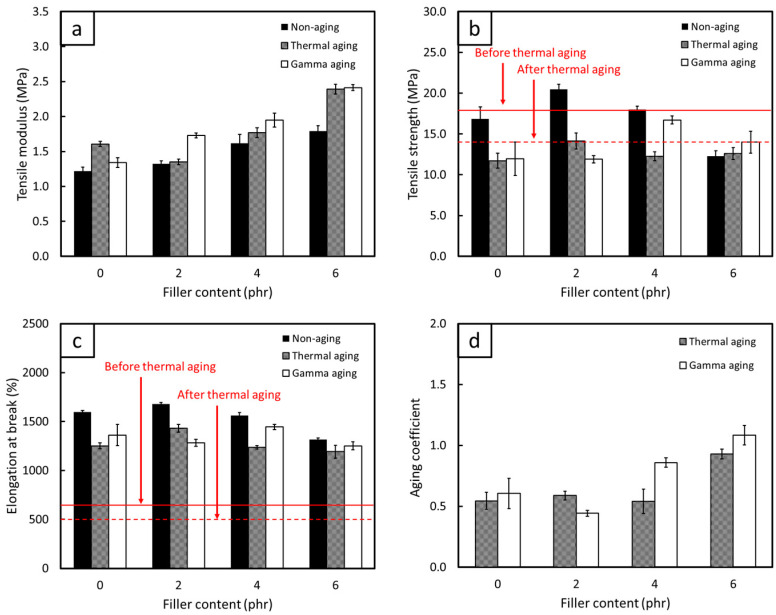
Mechanical properties, including (**a**) tensile modulus at 500% elongation, (**b**) tensile strength, (**c**) elongation at break, and (**d**) aging coefficient of thermal-aged and gamma-aged NRL gloves containing varying contents of DPP. The solid and dotted red lines in (**b**,**c**) represent the minimum requirements for non-aged and thermal-aged medical examination latex gloves, respectively, according to ASTM D3578-05. The error bars represent the standard deviations of the mean.

The comparisons of mechanical properties, consisting of TM500, TS, EB, and C_aging_, for non-aged, thermal-aged, and gamma-aged FPP/NRL gloves are shown in Figure 9, Figure 10 and Figure 11 (the results of statistical analysis for thermal-aged and gamma-aged FPP/NRL gloves are shown in Tables S9–S26). The results indicated that the TM500 values of the samples mostly increased after the aging process, probably due to the post-curing effect from heat and gamma irradiation that re-initiated the crosslinking of NR molecular chains, resulting in enhanced rigidity and TM500 values of the composites [58]. Notably, the thermal aging process initiated the post-curing (crosslinking) effects on the NRL gloves by enabling unused chemicals in the NR composites to be re-activated after the initial curing (during sample preparation), further increasing the overall rigidity of the composites [59]. On the other hand, gamma aging created free radicals in the NR matrix through the transfer of energy from the incident gamma radiation to the NR molecular chains, with the recombination of these polymer radicals leading to crosslinking and subsequently increasing the rigidity of the composites [32]. However, the TS and EB values tended to decrease for both aging conditions (except at some lower FPP contents, where the post-curing effect still dominated the aging process). This reduction in TS and EB after the aging could have been due to the initiation of a chain-scission mechanism that mostly occurred at the double bonds of C (C=C) in NR molecular chains (highly unsaturated hydrocarbon), subsequently lowering the strength and overall ability of the composites to withstand and elongate along external forces [60,61]. It should be noted that this chain-scission mechanism was, in fact, a competing factor to the crosslinking from post-curing, with the dominance of either mechanism largely defining the mechanical properties of the aged samples.

Figure 9, Figure 10 and Figure 11 reveal that the pristine NR suffered greater oxidative degradation from heat and radiation than any of the FPP/NRL gloves (95% confidence level), as evidenced by the lower C_aging_ in the former (0.54 for thermal aging and 0.61 for gamma aging) compared to values in the latter (Figure 9d, Figure 10d and Figure 11d). The lower C_aging_ in the pristine NR could have been due to the lack of antioxidants in the material formulation, which allowed free radicals created during the aging to initiate more chain-scission in NR molecular chains, substantially reducing the strength and flexibility of the aged composites. On the other hand, the addition of FPP, which had shown the ability to scavenge and stabilize free radicals (Section 3.1.2), could prevent or slow down oxidative degradation by acting as radical chain terminators that converted radicals into stable products [38,62]. Notably, the MPP/NRL gloves generally had higher C_aging_ values than those of the PPP/NRL and DPP/NRL gloves, with the C_aging_ values for the former being in the range of 0.85–1.01 for thermal aging and 0.87–1.12 for gamma aging. These higher C_aging_ values could have been due to the higher radical-scavenging capabilities of MPP compared to PPP and DPP, as seen by the much lower IC_50_ value of MPP (Table 2). This higher antioxidant ability of MPP limited chain-scission to dominate the aging process, subsequently preserving the mechanical properties of the aged composites. As a result, the investigations on the effects of thermal and gamma aging in this work clearly confirmed the great potential of utilizing MPP, PPP, and DPP as natural antioxidants in the production of NRL gloves, which could be used as a basis for the development of other rubber-based products.

### 3.3. Recommended FPP Contents for Actual Production

To determine the recommended FPP contents for the actual production of NRL gloves, the TS and EB values of the samples (both before and after thermal aging) were compared with the requirements of medical examination latex gloves, following the ASTM D3578-05 standard that requires the TS and EB values for non-aged (thermal-aged) latex gloves must be higher than 18 MPa and 650% (14 MPa and 500%), respectively. Based on the results shown in Figure 9, Figure 10 and Figure 11, the samples with higher TS and EB values than the requirements (shown as solid and dotted red lines for non-aged and thermal-aged samples, respectively) were those containing 2–4 phr of MPP, 4 phr of PPP, and 2 phr of DPP. These mentioned FPP contents could be regarded as the recommended contents for actual production, which would not only reinforce NRL gloves but also enhance the ability to resist oxidative degradation from heat and radiation, subsequently improving the useability and shelf-life of the materials. It should be noted that the pristine NR had TS and EB values lower than those required (both before and after thermal aging), implying their unsuitability as durable medical examination latex gloves as well as the necessity of using antioxidants in NR products.

## 4. Conclusions

This work developed NRL gloves containing FPPs, namely MPP, PPP, and DPP, as dual natural antioxidants and reinforcing bio-fillers, with varying FPP contents of 0, 2, 4, or 6 phr. The investigation on the thermal stability of the prepared FPPs using TGA showed that the decompositions of hemicellulose, cellulose, and lignin occurred at 200–220 °C, 315–330 °C, and 400–420 °C, respectively. In addition, MPP had the highest radical-scavenging capacity, as seen by its lowest IC_50_ value of 0.10 ± 0.01 mg/mL. The results also indicated that the FPPs acted as effective natural antioxidants and reinforcing bio-fillers to the NRL composites, as seen by their higher aging coefficients (under thermal and gamma aging) as well as the initial increases in tensile strength of the samples containing 2–4 phr of FPPs. Furthermore, by benchmarking the developed NRL gloves with the requirements of medical examination latex gloves (with and without thermal aging), according to ASTM D3578-05, the recommended FPP contents for actual use were 2–4 phr for MPP, 4 phr for PPP, and 2 phr for DPP. In conclusion, the overall results suggested that MPP, PPP, and DPP could be utilized as natural antioxidants and reinforcing bio-fillers for the production of NRL gloves that offered not only a simpler process to produce high-quality latex gloves but also sustainable methods to utilize agricultural and food wastes in rubber industries.

## Figures and Tables

**Table 1:** Material formulations of FPP/NRL gloves with their chemical names, contents, and roles [28].

Chemical	Content (phr)	Role
10% *w*/*w* fruit peel powder (FPP)	0, 2, 4, 6	Antioxidant/Reinforcing filler
10% *w*/*w* potassium hydroxide (KOH)	0.2	Stabilizer
10% *w*/*w* teric 16A16	0.02	Stabilizer
50% *w*/*w* sulfur (S)	0.8	Crosslinking agent
50% *w*/*w* zinc diethyl dithiocarbamate (ZDEC)	0.4	Accelerator
50% *w*/*w* zinc-2-mercaptobenzthiazole (ZMBT)	0.4	Accelerator
50% *w*/*w* zinc oxide (ZnO)	1.0	Activator
Distilled water (H_2_O)	170.5	Solvent

**Table 2:** Antioxidant activity (±standard deviation) at 50% radical-scavenging effect (IC_50_) of MPP, PPP, and DPP extracts, determined using Equations (4)–(6), respectively. The results of the statistical analysis of IC_50_ are shown in Table S1.

FPP Type	IC_50_ (mg/mL)
MPP	0.10 ± 0.01
PPP	6.08 ± 0.27
DPP	29.41 ± 0.78

**Table 3:** L*, a*, b*, and ΔE parameters of NRL gloves containing varying contents of MPP, PPP, and DPP. The numbers following ± represent standard deviations (S.D.) of the mean. The results for the statistical analysis of L*, a*, and b* parameters are shown in Tables S2–S4.

Sample	FPP Content (phr)	L*	a*	b*	ΔE
NRL	0	88.6 ± 0.1	0.2 ± 0.1	9.3 ± 0.1	–
MPP/NRL	2	72.0 ± 0.1	8.6 ± 0.1	31.2 ± 0.1	28.7
	4	66.5 ± 0.1	12.9 ± 0.1	40.1 ± 0.1	40.0
	6	57.6 ± 0.1	20.4 ± 0.1	47.5 ± 0.1	53.1
PPP/NRL	2	83.8 ± 0.1	0.6 ± 0.1	14.8 ± 0.1	7.3
	4	82.7 ± 0.1	0.4 ± 0.1	18.6 ± 0.1	11.0
	6	83.4 ± 0.1	0.4 ± 0.1	20.1 ± 0.1	12.0
DPP/NRL	2	83.7 ± 0.1	1.3 ± 0.1	16.1 ± 0.1	8.4
	4	79.3 ± 0.1	2.9 ± 0.1	22.0 ± 0.1	16.0
	6	79.6 ± 0.1	3.1 ± 0.1	24.5 ± 0.1	17.9

**Table 4:** Densities (±standard deviation) of NRL gloves containing varying contents of MPP, PPP, and DPP. The results of the statistical analysis of densities are shown in Table S5.

Sample	FPP Content (phr)	Density (g/cm^3^)
NRL	0	0.90 ± 0.02
MPP/NRL	2	0.97 ± 0.01
	4	0.98 ± 0.01
	6	0.98 ± 0.01
PPP/NRL	2	0.96 ± 0.01
	4	0.97 ± 0.01
	6	0.98 ± 0.01
DPP/NRL	2	0.96 ± 0.01
	4	0.97 ± 0.01
	6	0.99 ± 0.01

**Table 5:** Mechanical properties—tensile modulus at 500% elongation, tensile strength, and elongation at break of pristine NRL and NRL gloves containing varying contents of FPP. Values are shown as mean ± standard deviation. The results of the statistical analysis of mechanical properties for non-aged samples are shown in Tables S6–S8.

Sample	FPP Content (phr)	Tensile Modulus (MPa)	Tensile Strength (MPa)	Elongation at Break (%)
NRL	0	1.22 ± 0.06	16.85 ± 1.47	1599 ± 15
MPP/NRL	2	1.13 ± 0.09	18.10 ± 1.05	1700 ± 10
	4	1.71 ± 0.10	19.25 ± 0.91	1542 ± 11
	6	1.70 ± 0.10	15.94 ± 0.30	1512 ± 32
PPP/NRL	2	1.43 ± 0.01	15.12 ± 0.68	1478 ± 18
	4	1.36 ± 0.06	19.62 ± 0.68	1689 ± 14
	6	1.15 ± 0.11	16.14 ± 0.85	1647 ± 28
DPP/NRL	2	1.32 ± 0.04	20.48 ± 0.61	1680 ± 15
	4	1.62 ± 0.13	18.00 ± 0.40	1560 ± 32
	6	1.79 ± 0.08	12.29 ± 0.67	1316 ± 16

## Data Availability

The data presented in this study are available on request from the corresponding author.

## Supplementary Materials

The following supporting information can be downloaded at: https://www.mdpi.com/article/10.3390/antiox12051119/s1, Table S1: Statistical results based on the *t*-test for the determination of radical-scavenging effects (RSEs) of extracts from MPP, PPP, and DPP with varying extract concentrations. The values are shown as t(degrees of freedom) = t-value; *p* < *p*-value. Table S2: Statistical results based on the *t*-test for the determination of L* parameter for NRL gloves containing varying contents of MPP, PPP, and DPP. The values are shown as t(degrees of freedom) = t-value; *p* < *p*-value. Table S3: Statistical results based on the *t*-test for the determination of a* parameter for NRL gloves containing varying contents of MPP, PPP, and DPP. The values are shown as t(degrees of freedom) = t-value; *p* < *p*-value. Table S4: Statistical results based on the *t*-test for the determination of b* parameter for NRL gloves containing varying contents of MPP, PPP, and DPP. The values are shown as t(degrees of freedom) = t-value; *p* < *p*-value. Table S5: Statistical results based on the *t*-test for the determination of densities for NRL gloves containing varying contents of MPP, PPP, and DPP. The values are shown as t(degrees of freedom) = t-value; *p* < *p*-value. Table S6: Statistical results based on the *t*-test for the determination of tensile strength for non-aged NRL gloves containing varying contents of MPP, PPP, and DPP. The values are shown as t(degrees of freedom) = t-value; *p* < *p*-value. Table S7: Statistical results based on the *t*-test for the determination of tensile modulus for non-aged NRL gloves containing varying contents of MPP, PPP, and DPP. The values are shown as t(degrees of freedom) = t-value; *p* < *p*-value. Table S8: Statistical results based on the *t*-test for the determination of elongation at break for non-aged NRL gloves containing varying contents of MPP, PPP, and DPP. The values are shown as t(degrees of freedom) = t-value; *p* < *p*-value. Table S9: Statistical results based on the *t*-test for the determination of tensile strength for thermal-aged NRL gloves containing varying contents of MPP. The values are shown as t(degrees of freedom) = t-value; *p* < *p*-value. Table S10: Statistical results based on the *t*-test for the determination of tensile strength for gamma-aged NRL gloves containing varying contents of MPP. The values are shown as t(degrees of freedom) = t-value; *p* < *p*-value. Table S11: Statistical results based on the *t*-test for the determination of tensile strength for thermal-aged NRL gloves containing varying contents of PPP. The values are shown as t(degrees of freedom) = t-value; *p* < *p*-value. Table S12: Statistical results based on the *t*-test for the determination of tensile strength for gamma-aged NRL gloves containing varying contents of PPP. The values are shown as t(degrees of freedom) = t-value; *p* < *p*-value. Table S13: Statistical results based on the *t*-test for the determination of tensile strength for thermal-aged NRL gloves containing varying contents of DPP. The values are shown as t(degrees of freedom) = t-value; *p* < *p*-value. Table S14: Statistical results based on the *t*-test for the determination of tensile strength for gamma-aged NRL gloves containing varying contents of DPP. The values are shown as t(degrees of freedom) = t-value; *p* < *p*-value. Table S15: Statistical results based on the *t*-test for the determination of tensile modulus for thermal-aged NRL gloves containing varying contents of MPP. The values are shown as t(degrees of freedom) = t-value; *p* < *p*-value. Table S16: Statistical results based on the *t*-test for the determination of tensile modulus for gamma-aged NRL gloves containing varying contents of MPP. The values are shown as t(degrees of freedom) = t-value; *p* < *p*-value. Table S17: Statistical results based on the *t*-test for the determination of tensile modulus for thermal-aged NRL gloves containing varying contents of PPP. The values are shown as t(degrees of freedom) = t-value; *p* < *p*-value. Table S18: Statistical results based on the *t*-test for the determination of tensile modulus for gamma-aged NRL gloves containing varying contents of PPP. The values are shown as t(degrees of freedom) = t-value; *p* < *p*-value. Table S19: Statistical results based on the *t*-test for the determination of tensile modulus for thermal-aged NRL gloves containing varying contents of DPP. The values are shown as t(degrees of freedom) = t-value; *p* < *p*-value. Table S20: Statistical results based on the *t*-test for the determination of tensile modulus for gamma-aged NRL gloves containing varying contents of DPP. The values are shown as t(degrees of freedom) = t-value; *p* < *p*-value. Table S21: Statistical results based on the *t*-test for the determination of elongation at break for thermal-aged NRL gloves containing varying contents of MPP. The values are shown as t(degrees of freedom) = t-value; *p* < *p*-value. Table S22: Statistical results based on the *t*-test for the determination of elongation at break for gamma-aged NRL gloves containing varying contents of MPP. The values are shown as t(degrees of freedom) = t-value; *p* < *p*-value. Table S23: Statistical results based on the *t*-test for the determination of elongation at break for thermal-aged NRL gloves containing varying contents of PPP. The values are shown as t(degrees of freedom) = t-value; *p* < *p*-value. Table S24: Statistical results based on the *t*-test for the determination of elongation at break for gamma-aged NRL gloves containing varying contents of PPP. The values are shown as t(degrees of freedom) = t-value; *p* < *p*-value. Table S25: Statistical results based on the *t*-test for the determination of elongation at break for thermal-aged NRL gloves containing varying contents of DPP. The values are shown as t(degrees of freedom) = t-value; *p* < *p*-value. Table S26: Statistical results based on the *t*-test for the determination of elongation at break for gamma-aged NRL gloves containing varying contents of DPP. The values are shown as t(degrees of freedom) = t-value; *p* < *p*-value.
