# Supplementary material for: Fruit Peel Powder as Natural Antioxidant and Reinforcing Bio-Filler in Natural Rubber Latex Gloves: Cases of Mangosteen, Pomelo and Durian
Source: Antioxidants (Basel). 2023 May 18;12(5):1119. doi: 10.3390/antiox12051119 (PMC10215814; doi:10.3390/antiox12051119)
Supplement: Supplementary file 1 [file antioxidants-12-01119-s001.zip › antioxidants-2389452-supplementary.pdf]

Supplementary Materials

Fruit Peel Powder as Natural Antioxidant and Reinforcing Bio-Filler in Natural Rubber Latex Gloves: Cases of Mangosteen, Pomelo and Durian

Arkarapol Thumwong [1,2], Jitsuna Darachai [2], Nuatawan Thamrongsiripak [3], Shinji Tokonami [4], Tetsuo Ishikawa [5] and Kiadtisak Saenboonruang [2,6,7,8,*]

1 Department of Materials Science, Faculty of Science, Kasetsart University, Bangkok 10900, Thailand; arkarapol.th@ku.th
2 Special Research Unit of Radiation Technology for Advanced Materials (RTAM), Faculty of Science, Kasetsart University, Bangkok 10900, Thailand; jitsuna.d@ku.th
3 Thailand Institute of Nuclear Technology (Public Organization), Nakhorn Nayok 26120, Thailand; nuatawan@tint.or.th
4 Institute of Radiation Emergency Medicine, Hirosaki University, Aomori 0368564, Japan; tokonami@hirosaki-u.ac.jp
5 Department of Radiation Physics and Chemistry, Fukushima Medical University, Fukushima 9601295, Japan; isikawat@fmu.ac.jp
6 Department of Applied Radiation and Isotopes, Faculty of Science, Kasetsart University, Bangkok 10900, Thailand
7 Kasetsart University Research and Development Institute, Kasetsart University, Bangkok 10900, Thailand
8 Specialized Center of Rubber and Polymer Materials in Agriculture and Industry (RPM), Faculty of Science, Kasetsart University, Bangkok 10900, Thailand
* Correspondence: kiadtisak.s@ku.th; Tel.: +66-2562-5555 (ext. 646219)

Table S1. Statistical results based on the t-test for the determination of radical scavenging effects (RSEs) of extracts from MPP, PPP, and DPP with varying extract concentrations. The values are shown as t(degrees of freedom) = t-value; p < p-value.

	MPP	PPP	DPP
MPP	–	t(3) = -38.61; p < 0.0001	t(3) = -64.95; p < 0.0001
PPP	t(3) = 38.61; p < 0.0001	–	t(3) = -77.28; p < 0.0001
DPP	t(3) = 64.95; p < 0.0001	t(3) = 77.28; p < 0.0001	–

Table S2. Statistical results based on the t-test for the determination of *L** parameter for NRL gloves containing varying contents of MPP, PPP, and DPP. The values are shown as t(degrees of freedom) = t-value; p < p-value.

	Pristine NR	2-phr MPP	4-phr MPP	6-phr MPP	2-phr PPP	4-phr PPP	6-phr PPP	2-phr DPP	4-phr DPP	6-phr DPP
**Pristine NR**	–	t(3) = 1084.11; p < 0.0001	t(3) = 944.86; p < 0.0001	t(3) = 668.20; p < 0.0001	t(3) = 56.56; p < 0.0002	t(3) = 146.48; p < 0.0001	t(3) = 148.46; p < 0.0001	t(3) = 486.00; p < 0.0001	t(3) = 133.95; p < 0.0001	t(3) = 257.79; p < 0.0001
**2-phr MPP**	t(3) = -1084.11; p < 0.0001	–	t(3) = 622.13; p < 0.0001	t(3) = 438.12; p < 0.0001	t(3) = -162.94; p < 0.0001	t(3) = -402.15; p < 0.0001	t(3) = -561.92; p < 0.0001	t(3) = -506.62; p < 0.0001	t(3) = -132.18; p < 0.0001	t(3) = -366.53; p < 0.0001
**4-phr MPP**	t(3) = -944.86; p < 0.0001	t(3) = -622.13; p < 0.0001	–	t(3) = 370.12; p < 0.0001	t(3) = -272.16; p < 0.0001	t(3) = -914.30; p < 0.0001	t(3) = -1461.85; p < 0.0001	t(3) = -540.50; p < 0.0001	t(3) = -275.69; p < 0.0001	t(3) = 1091.37; p < 0.0001
**6-phr MPP**	t(3) = -668.20; p < 0.0001	t(3) = -438.12; p < 0.0001	t(3) = -370.12; p < 0.0001	–	t(3) = -664.98; p < 0.0001	t(3) = -3753.50; p < 0.0001	t(3) = -1933.25; p < 0.0001	t(3) = -470.16; p < 0.0001	t(3) = -928.43; p < 0.0001	t(3) = -1831.62; p < 0.0001
**2-phr PPP**	t(3) = -56.56; p < 0.0002	t(3) = 162.94; p < 0.0001	t(3) = 272.16; p < 0.0001	t(3) = 664.98; p < 0.0001	–	t(3) = 24.22; p < 0.0008	t(3) = 6.81; p < 0.0104	t(3) = -0.53; p < 0.3248	t(3) = -214.73; p < 0.0001	t(3) = -80.29; p < 0.0001
**4-phr PPP**	t(3) = -146.48; p < 0.0001	t(3) = 402.15; p < 0.0001	t(3) = 914.30; p < 0.0001	t(3) = 3753.50; p < 0.0001	t(3) = -24.22; p < 0.0008	–	t(3) = -113.00; p < 0.0001	t(3) = 21.49; p < 0.0011	t(3) = -116.39; p < 0.0001	t(3) = -521.35; p < 0.0001
**6-phr PPP**	t(3) = -148.46; p < 0.0001	t(3) = 561.92; p < 0.0001	t(3) = 1461.85; p < 0.0001	t(3) = 1933.25; p < 0.0001	t(3) = -6.81; p < 0.0104	t(3) = 113.00; p < 0.0001	–	t(3) = 7.08; p < 0.0097	t(3) = -118.20; p < 0.0001	t(3) = -1129.00; p < 0.0001
**2-phr DPP**	t(3) = -486.00; p < 0.0001	t(3) = 506.62; p < 0.0001	t(3) = 540.50; p < 0.0001	t(3) = 470.16; p < 0.0001	t(3) = 0.53; p < 0.3248	t(3) = -21.49; p < 0.0011	t(3) = -7.08; p < 0.0097	–	t(3) = 56.59; p < 0.0002	t(3) = 93.37; p < 0.0001
**4-phr DPP**	t(3) = -133.95; p < 0.0001	t(3) = 132.18; p < 0.0001	t(3) = 275.69; p < 0.0001	t(3) = 928.43; p < 0.0001	t(3) = 214.73; p < 0.0001	t(3) = 116.39; p < 0.0001	t(3) = 118.20; p < 0.0001	t(3) = -56.59; p < 0.0002	–	t(3) = -10.10; p < 0.0048
**6-phr DPP**	t(3) = -257.79; p < 0.0001	t(3) = 366.53; p < 0.0001	t(3) = -1091.37; p < 0.0001	t(3) = 1831.62; p < 0.0001	t(3) = 80.29; p < 0.0001	t(3) = 521.35; p < 0.0001	t(3) = 1129.00; p < 0.0001	t(3) = -93.37; p < 0.0001	t(3) = 10.10; p < 0.0048	–

Table S3. Statistical results based on the t-test for the determination of *a** parameter for NRL gloves containing varying contents of MPP, PPP, and DPP. The values are shown as t(degrees of freedom) = t-value; p < p-value.

	**Pristine NR**	**2-phr MPP**	**4-phr MPP**	**6-phr MPP**	**2-phr PPP**	**4-phr PPP**	**6-phr PPP**	**2-phr DPP**	**4-phr DPP**	**6-phr DPP**
**Pristine NR**	–	t(3) = -831.00; p < 0.0001	t(3) = -2183.31; p < 0.0001	t(3) = -872.52; p < 0.0001	t(3) = -58.00; p < 0.0001	t(3) = -52.00; p < 0.0002	t(3) = -15.50; p < 0.0021	t(3) = -58.01; p < 0.0001	t(3) = -398.00; p < 0.0001	t(3) = -319.38; p < 0.0001
**2-phr MPP**	t(3) = 831.00; p < 0.0001	–	t(3) = 831.00; p < 0.0001	t(3) = 775.11; p < 0.0001	t(3) = 898.42; p < 0.0001	t(3) = 922.61; p < 0.0001	t(3) = 399.65; p < 0.0001	t(3) = 300.92; p < 0.0001	t(3) = 389.32; p < 0.0001	t(3) = 1648.00; p < 0.0001
**4-phr MPP**	t(3) = 2183.31; p < 0.0001	t(3) = -831.00; p < 0.0001	–	t(3) = -433.59; p < 0.0001	t(3) = 3676.00; p < 0.0001	t(3) = 3740.00; p < 0.0001	t(3) = 669.93; p < 0.0001	t(3) = 623.05; p < 0.0001	t(3) = 1132.38; p < 0.0001	t(3) = 2947.00; p < 0.0001
**6-phr MPP**	t(3) = 872.52; p < 0.0001	t(3) = -775.11; p < 0.0001	t(3) = 433.59; p < 0.0001	–	t(3) = 1120.48; p < 0.0001	t(3) = 985.24; p < 0.0001	t(3) = 565.34; p < 0.0001	t(3) = 699.05; p < 0.0001	t(3) = 727.91; p < 0.0001	t(3) = 1192.96; p < 0.0001
**2-phr PPP**	t(3) = 58.00; p < 0.0001	t(3) = -898.42; p < 0.0001	t(3) = -3676.00; p < 0.0001	t(3) = -1120.48; p < 0.0001	–	t(3) = 64.00; p < 0.0008	t(3) = 9.00; p < 0.0061	t(3) = 45.17; p < 0.0002	t(3) = 340.00; p < 0.0001	t(3) = 420.89; p < 0.0001
**4-phr PPP**	t(3) = 52.00; p < 0.0002	t(3) = -922.61; p < 0.0001	t(3) = -3740.00; p < 0.0001	t(3) = -985.24; p < 0.0001	t(3) = -64.00; p < 0.0008	–	t(3) = -2.00; p < 0.0918	t(3) = 54.20; p < 0.0002	t(3) = 429.55; p < 0.0001	t(3) = 396.50; p < 0.0001
**6-phr PPP**	t(3) = 15.50; p < 0.0021	t(3) = -399.65; p < 0.0001	t(3) = -669.93; p < 0.0001	t(3) = -565.34; p < 0.0001	t(3) = -9.00; p < 0.0061	t(3) = 2.00; p < 0.0918	–	t(3) = 30.14; p < 0.0005	t(3) = 138.71; p < 0.0001	t(3) = 125.38; p < 0.0001
**2-phr DPP**	t(3) = 58.01; p < 0.0001	t(3) = -300.92; p < 0.0001	t(3) = -623.05; p < 0.0001	t(3) = -699.05; p < 0.0001	t(3) = -45.17; p < 0.0002	t(3) = -54.20; p < 0.0002	t(3) = -30.14; p < 0.0005	–	t(3) = -131.19; p < 0.0002	t(3) = -83.59; p < 0.0001
**4-phr DPP**	t(3) = 398.00; p < 0.0001	t(3) = -389.32; p < 0.0001	t(3) = -1132.38; p < 0.0001	t(3) = -727.91; p < 0.0001	t(3) = -340.00; p < 0.0001	t(3) = -429.55; p < 0.0001	t(3) = -30.14; p < 0.0005	t(3) = 131.19; p < 0.0002	–	t(3) = -13.59; p < 0.0027
**6-phr DPP**	t(3) = 319.38; p < 0.0001	t(3) = -1648.00; p < 0.0001	t(3) = -2947.00; p < 0.0001	t(3) = -1192.96; p < 0.0001	t(3) = -420.89; p < 0.0001	t(3) = -396.50; p < 0.0001	t(3) = -125.38; p < 0.0001	t(3) = 83.59; p < 0.0001	t(3) = 13.59; p < 0.0027	–

Table S4. Statistical results based on the t-test for the determination of *b** parameter for NRL gloves containing varying contents of MPP, PPP, and DPP. The values are shown as t(degrees of freedom) = t-value; p < p-value.

	Pristine NR	2-phr MPP	4-phr MPP	6-phr MPP	2-phr PPP	4-phr PPP	6-phr PPP	2-phr DPP	4-phr DPP	6-phr DPP
**Pristine NR**	–	t(3) = -1052.04; p < 0.0001	t(3) = -2565.49; p < 0.0001	t(3) = -757.76; p < 0.0001	t(3) = -621.75; p < 0.0001	t(3) = -1389.50; p < 0.0001	t(3) = -1227.25; p < 0.0001	t(3) = -327.62; p < 0.0001	t(3) = -764.20; p < 0.0001	t(3) = -1267.77; p < 0.0001
**2-phr MPP**	t(3) = 1052.04; p < 0.0001	–	t(3) = -743.30; p < 0.0001	t(3) = -304.28; p < 0.0001	t(3) = 751.06; p < 0.0001	t(3) = 463.14; p < 0.0001	t(3) = 425.47; p < 0.0001	t(3) = 724.42; p < 0.0001	t(3) = 309.29; p < 0.0001	t(3) = 233.97; p < 0.0001
**4-phr MPP**	t(3) = 2565.49; p < 0.0001	t(3) = 743.30; p < 0.0001	–	t(3) = -163.89; p < 0.0001	t(3) = 2535.00; p < 0.0001	t(3) = 1245.34; p < 0.0001	t(3) = 1000.50; p < 0.0001	t(3) = 1998.03; p < 0.0001	t(3) = 1025.98; p < 0.0001	t(3) = 935.80; p < 0.0001
**6-phr MPP**	t(3) = 757.76; p < 0.0001	t(3) = 304.28; p < 0.0001	t(3) = 163.89; p < 0.0001	–	t(3) = 786.39; p < 0.0001	t(3) = 587.08; p < 0.0001	t(3) = 467.62; p < 0.0001	t(3) = 942.10; p < 0.0001	t(3) = 732.35; p < 0.0001	t(3) = 566.85; p < 0.0001
**2-phr PPP**	t(3) = 621.75; p < 0.0001	t(3) = -751.06; p < 0.0001	t(3) = -2535.00; p < 0.0001	t(3) = -786.39; p < 0.0001	–	t(3) = -378.00; p < 0.0001	t(3) = -308.31; p < 0.0001	t(3) = 100.25; p < 0.0001	t(3) = 822.45; p < 0.0001	t(3) = 1463.00; p < 0.0001
**4-phr PPP**	t(3) = 1389.50; p < 0.0001	t(3) = -463.14; p < 0.0001	t(3) = -1245.34; p < 0.0001	t(3) = -587.08; p < 0.0001	t(3) = 378.00; p < 0.0001	–	t(3) = -156.00; p < 0.0001	t(3) = -104.71; p < 0.0001	t(3) = 239.05; p < 0.0001	t(3) = 677.31; p < 0.0001
**6-phr PPP**	t(3) = 1227.25; p < 0.0001	t(3) = -425.47; p < 0.0001	t(3) = -1000.50; p < 0.0001	t(3) = -467.62; p < 0.0001	t(3) = 308.31; p < 0.0001	t(3) = 156.00; p < 0.0001	–	t(3) = -135.12; p < 0.0001	t(3) = 79.60; p < 0.0001	t(3) = 237.80; p < 0.0001
**2-phr DPP**	t(3) = 327.62; p < 0.0001	t(3) = -724.42; p < 0.0001	t(3) = -1998.03; p < 0.0001	t(3) = -942.10; p < 0.0001	t(3) = -100.25; p < 0.0001	t(3) = 104.71; p < 0.0001	t(3) = 135.12; p < 0.0001	–	t(3) = -407.21; p < 0.0001	t(3) = -477.18; p < 0.0001
**4-phr DPP**	t(3) = 764.20; p < 0.0001	t(3) = -309.29; p < 0.0001	t(3) = -1025.98; p < 0.0001	t(3) = -732.35; p < 0.0001	t(3) = -822.45; p < 0.0001	t(3) = -239.05; p < 0.0001	t(3) = -79.60; p < 0.0001	t(3) = 407.21; p < 0.0001	–	t(3) = -433.01; p < 0.0001
**6-phr DPP**	t(3) = 1267.77; p < 0.0001	t(3) = -233.97; p < 0.0001	t(3) = -935.80; p < 0.0001	t(3) = -566.85; p < 0.0001	t(3) = -1463.00; p < 0.0001	t(3) = -677.31; p < 0.0001	t(3) = -237.80; p < 0.0001	t(3) = 477.18; p < 0.0001	t(3) = 433.01; p < 0.0001	–

Table S5. Statistical results based on the t-test for the determination of densities for NRL gloves containing varying contents of MPP, PPP, and DPP. The values are shown as t(degrees of freedom) = t-value; p < p-value.

	**Pristine NR**	**2-phr MPP**	**4-phr MPP**	**6-phr MPP**	**2-phr PPP**	**4-phr PPP**	**6-phr PPP**	**2-phr DPP**	**4-phr DPP**	**6-phr DPP**
**Pristine NR**	–	t(3) = -6.26; p < 0.0123	t(3) = -6.26; p < 0.0123	t(3) = -6.26; p < 0.0123	t(3) = -6.26; p < 0.0123	t(3) = -6.26; p < 0.0123	t(3) = -7.05; p < 0.0098	t(3) = -5.51; p < 0.0157	t(3) = -8.68; p < 0.0065	t(3) = -9.59; p < 0.0054
**2-phr MPP**	t(3) = 6.26; p < 0.0123	–	t(3) = -2.76; p < 0.0551	t(3) = -1.68; p < 0.1173	t(3) = 2.87; p < 0.0516	t(3) = 4.35; p < 0.0245	t(3) = -1.98; p < 0.0934	t(3) = 3.67; p < 0.0335	t(3) = 0.63; p < 0.2957	t(3) = -2.02; p < 0.0907
**4-phr MPP**	t(3) = 6.26; p < 0.0123	t(3) = 2.76; p < 0.0551	–	t(3) = 0.00; p < 0.5000	t(3) = 3.50; p < 0.0364	t(3) = 8.17; p < 0.0073	t(3) = 2.43; p < 0.0679	t(3) = 7.45; p < 0.0088	t(3) = 2.56; p < 0.0623	t(3) = -0.85; p < 0.2415
**6-phr MPP**	t(3) = 6.26; p < 0.0123	t(3) = 1.68; p < 0.1173	t(3) = 0.00; p < 0.5000	–	t(3) = 2.24; p < 0.0771	t(3) = 4.49; p < 0.0231	t(3) = 1.51; p < 0.1352	t(3) = 6.86; p < 0.0103	t(3) = 4.16; p < 0.0266	t(3) = -5.20; p < 0.0175
**2-phr PPP**	t(3) = 6.26; p < 0.0123	t(3) = -2.87; p < 0.0516	t(3) = -3.50; p < 0.0364	t(3) = -2.24; p < 0.0771	–	t(3) = -0.15; p < 0.4464	t(3) = -2.62; p < 0.0601	t(3) = 0.46; p < 0.3450	t(3) = -0.24; p < 0.4148	t(3) = -2.46; p < 0.0667
**4-phr PPP**	t(3) = 6.26; p < 0.0123	t(3) = -4.35; p < 0.0245	t(3) = -8.17; p < 0.0073	t(3) = -4.49; p < 0.0231	t(3) = 0.15; p < 0.4464	–	t(3) = -34.00; p < 0.0004	t(3) = 2.29; p < 0.0744	t(3) = -0.28; p < 0.4041	t(3) = -4.59; p < 0.0222
**6-phr PPP**	t(3) = 7.05; p < 0.0098	t(3) = 1.98; p < 0.0934	t(3) = -2.43; p < 0.0679	t(3) = -1.51; p < 0.1352	t(3) = 2.62; p < 0.0601	t(3) = 34.00; p < 0.0004	–	t(3) = 12.20; p < 0.0033	t(3) = 1.28; p < 0.1638	t(3) = -2.00; p < 0.0918
**2-phr DPP**	t(3) = 5.51; p < 0.0157	t(3) = -3.67; p < 0.0335	t(3) = -7.45; p < 0.0088	t(3) = -6.86; p < 0.0103	t(3) = -0.46; p < 0.3450	t(3) = -2.29; p < 0.0744	t(3) = -12.20; p < 0.0033	–	t(3) = -0.81; p < 0.2510	t(3) = -6.61; p < 0.0111
**4-phr DPP**	t(3) = 8.68; p < 0.0065	t(3) = -0.63; p < 0.2957	t(3) = -2.56; p < 0.0623	t(3) = -4.16; p < 0.0266	t(3) = 0.24; p < 0.4148	t(3) = 0.28; p < 0.4041	t(3) = -1.28; p < 0.1638	t(3) = 0.81; p < 0.2510	–	t(3) = -5.89; p < 0.0138
**6-phr DPP**	t(3) = 9.59; p < 0.0054	t(3) = 2.02; p < 0.0907	t(3) = 0.85; p < 0.2415	t(3) = 5.20; p < 0.0175	t(3) = 2.46; p < 0.0667	t(3) = 4.59; p < 0.0222	t(3) = 2.00; p < 0.0918	t(3) = 6.61; p < 0.0111	t(3) = 5.89; p < 0.0138	–

Table S6. Statistical results based on the t-test for the determination of tensile strength for non-aged NRL gloves containing varying contents of MPP, PPP, and DPP. The values are shown as t(degrees of freedom) = t-value; p < p-value.

	Pristine NR	2-phr MPP	4-phr MPP	6-phr MPP	2-phr PPP	4-phr PPP	6-phr PPP	2-phr DPP	4-phr DPP	6-phr DPP
**Pristine NR**	–	t(3) = -1.02; p < 0.2083	t(3) = -2.79; p < 0.0539	t(3) = 1.10; p < 0.1922	t(3) = 2.20; p < 0.0794	t(3) = -5.91; p < 0.0137	t(3) = 1.40; p < 0.1485	t(3) = -6.31; p < 0.0121	t(3) = -1.22; p < 0.1739	t(3) = 4.48; p < 0.0232
**2-phr MPP**	t(3) = 1.02; p < 0.2083	–	t(3) = -2.76; p < 0.0551	t(3) = -2.78; p < 0.0543	t(3) = 2.99; p < 0.0479	t(3) = -1.70; p < 0.1157	t(3) = 2.68; p < 0.0580	t(3) = -2.60; p < 0.0609	t(3) = 0.28; p < 0.4017	t(3) = 6.21; p < 0.0125
**4-phr MPP**	t(3) = 2.79; p < 0.0539	t(3) = 2.76; p < 0.0551	–	t(3) = 4.88; p < 0.0197	t(3) = 4.79; p < 0.0205	t(3) = -0.60; p < 0.3041	t(3) = 8.68; p < 0.0065	t(3) = -1.78; p < 0.1086	t(3) = 3.52; p < 0.0361	t(3) = 7.64; p < 0.0084
**6-phr MPP**	t(3) = -1.10; p < 0.1922	t(3) = 2.78; p < 0.0543	t(3) = -4.88; p < 0.0197	–	t(3) = 3.73; p < 0.0324	t(3) = -10.38; p < 0.0046	t(3) = -0.36; p < 0.3782	t(3) = -17.76; p < 0.0016	t(3) = -5.05; p < 0.0185	t(3) = 14.52; p < 0.0024
**2-phr PPP**	t(3) = -2.20; p < 0.0794	t(3) = -2.99; p < 0.0479	t(3) = -4.79; p < 0.0205	t(3) = -3.73; p < 0.0324	–	t(3) = -11.96; p < 0.0035	t(3) = -1.52; p < 0.1339	t(3) = -22.31; p < 0.0010	t(3) = -4.61; p < 0.0220	t(3) = 11.38; p < 0.0038
**4-phr PPP**	t(3) = 5.91; p < 0.0137	t(3) = 1.70; p < 0.1157	t(3) = 0.60; p < 0.3041	t(3) = 10.38; p < 0.0046	t(3) = 11.96; p < 0.0035	–	t(3) = 10.65; p < 0.0043	t(3) = -6.32; p < 0.0121	t(3) = 3.00; p < 0.0476	t(3) = 12.81; p < 0.0030
**6-phr PPP**	t(3) = -1.40; p < 0.1485	t(3) = -2.68; p < 0.0580	t(3) = -8.68; p < 0.0065	t(3) = 0.36; p < 0.3782	t(3) = 1.52; p < 0.1339	t(3) = -10.65; p < 0.0043	–	t(3) = -9.71; p < 0.0052	t(3) = -3.83; p < 0.0310	t(3) = 4.73; p < 0.0209
**2-phr DPP**	t(3) = 6.31; p < 0.0121	t(3) = 2.60; p < 0.0609	t(3) = 1.78; p < 0.1086	t(3) = 17.76; p < 0.0016	t(3) = 22.31; p < 0.0010	t(3) = 6.32; p < 0.0121	t(3) = 9.71; p < 0.0052	–	t(3) = 4.57; p < 0.0224	t(3) = 18.36; p < 0.0015
**4-phr DPP**	t(3) = 1.22; p < 0.1739	t(3) = -0.28; p < 0.4017	t(3) = -3.52; p < 0.0361	t(3) = 5.05; p < 0.0185	t(3) = 4.61; p < 0.0220	t(3) = -3.00; p < 0.0476	t(3) = 3.83; p < 0.0310	t(3) = -4.57; p < 0.0224	–	t(3) = 9.54; p < 0.0054
**6-phr DPP**	t(3) = -4.48; p < 0.0232	t(3) = -6.21; p < 0.0125	t(3) = -7.64; p < 0.0084	t(3) = -14.52; p < 0.0024	t(3) = -11.38; p < 0.0038	t(3) = -12.81; p < 0.0030	t(3) = -4.73; p < 0.0209	t(3) = -18.36; p < 0.0015	t(3) = -9.54; p < 0.0054	–

Table S7. Statistical results based on the t-test for the determination of tensile modulus for non-aged NRL gloves containing varying contents of MPP, PPP, and DPP. The values are shown as t(degrees of freedom) = t-value; $p <$ p-value.

	**Pristine NR**	**2-phr MPP**	**4-phr MPP**	**6-phr MPP**	**2-phr PPP**	**4-phr PPP**	**6-phr PPP**	**2-phr DPP**	**4-phr DPP**	**6-phr DPP**
**Pristine NR**	–	$t(3) = 1.37$; $p < 0.1518$	$t(3) = -14.58$; $p < 0.0023$	$t(3) = -13.70$; $p < 0.0026$	$t(3) = -7.11$; $p < 0.0096$	$t(3) = -2.55$; $p < 0.0628$	$t(3) = 1.26$; $p < 0.1680$	$t(3) = -3.97$; $p < 0.0290$	$t(3) = -4.16$; $p < 0.0266$	$t(3) = -8.57$; $p < 0.0067$
**2-phr MPP**	$t(3) = -1.37$; $p < 0.1518$	–	$t(3) = -5.87$; $p < 0.0139$	$t(3) = -5.70$; $p < 0.0147$	$t(3) = -6.04$; $p < 0.0132$	$t(3) = -2.85$; $p < 0.0522$	$t(3) = -0.15$; $p < 0.4467$	$t(3) = -2.68$; $p < 0.0579$	$t(3) = -4.45$; $p < 0.0235$	$t(3) = -45.65$; $p < 0.0002$
**4-phr MPP**	$t(3) = 14.58$; $p < 0.0023$	$t(3) = 5.87$; $p < 0.0139$	–	$t(3) = 1.89$; $p < 0.0997$	$t(3) = 4.96$; $p < 0.0192$	$t(3) = 5.43$; $p < 0.161$	$t(3) = 12.14$; $p < 0.0034$	$t(3) = 9.65$; $p < 0.0053$	$t(3) = 0.93$; $p < 0.2244$	$t(3) = -0.80$; $p < 0.2538$
**6-phr MPP**	$t(3) = 13.70$; $p < 0.0026$	$t(3) = 5.70$; $p < 0.0147$	$t(3) = -1.89$; $p < 0.0997$	–	$t(3) = 4.90$; $p < 0.0196$	$t(3) = 5.80$; $p < 0.0142$	$t(3) = 14.53$; $p < 0.0024$	$t(3) = 10.58$; $p < 0.0044$	$t(3) = 0.84$; $p < 0.2450$	$t(3) = -0.98$; $p < 0.2154$
**2-phr PPP**	$t(3) = 7.11$; $p < 0.0096$	$t(3) = 6.04$; $p < 0.0132$	$t(3) = -4.96$; $p < 0.0192$	$t(3) = -4.90$; $p < 0.0196$	–	$t(3) = 1.94$; $p < 0.0959$	$t(3) = 4.60$; $p < 0.0220$	$t(3) = 4.43$; $p < 0.0237$	$t(3) = -2.53$; $p < 0.0635$	$t(3) = -8.18$; $p < 0.0073$
**4-phr PPP**	$t(3) = 2.55$; $p < 0.0628$	$t(3) = 2.85$; $p < 0.0522$	$t(3) = -5.43$; $p < 0.161$	$t(3) = -5.80$; $p < 0.0142$	$t(3) = -1.94$; $p < 0.0959$	–	$t(3) = 5.25$; $p < 0.0172$	$t(3) = 1.17$; $p < 0.1812$	$t(3) = -6.24$; $p < 0.0123$	$t(3) = -6.19$; $p < 0.0126$
**6-phr PPP**	$t(3) = -1.26$; $p < 0.1680$	$t(3) = 0.15$; $p < 0.4467$	$t(3) = -12.14$; $p < 0.0034$	$t(3) = -14.53$; $p < 0.0024$	$t(3) = -4.60$; $p < 0.0220$	$t(3) = -5.25$; $p < 0.0172$	–	$t(3) = -4.60$; $p < 0.0221$	$t(3) = -7.31$; $p < 0.0091$	$t(3) = -6.21$; $p < 0.00125$
**2-phr DPP**	$t(3) = 3.97$; $p < 0.0290$	$t(3) = 2.68$; $p < 0.0579$	$t(3) = -9.65$; $p < 0.0053$	$t(3) = -10.58$; $p < 0.0044$	$t(3) = -4.43$; $p < 0.0237$	$t(3) = -1.17$; $p < 0.1812$	$t(3) = 4.60$; $p < 0.0221$	–	$t(3) = -4.20$; $p < 0.0261$	$t(3) = -6.90$; $p < 0.0102$
**4-phr DPP**	$t(3) = 4.16$; $p < 0.0266$	$t(3) = 4.45$; $p < 0.0235$	$t(3) = -0.93$; $p < 0.2244$	$t(3) = -0.84$; $p < 0.2450$	$t(3) = 2.53$; $p < 0.0635$	$t(3) = 6.24$; $p < 0.0123$	$t(3) = 7.31$; $p < 0.0091$	$t(3) = 4.20$; $p < 0.0261$	–	$t(3) = -1.82$; $p < 0.1048$
**6-phr DPP**	$t(3) = 8.57$; $p < 0.0067$	$t(3) = 45.65$; $p < 0.0002$	$t(3) = 0.80$; $p < 0.2538$	$t(3) = 0.98$; $p < 0.2154$	$t(3) = 8.18$; $p < 0.0073$	$t(3) = 6.19$; $p < 0.0126$	$t(3) = 6.21$; $p < 0.00125$	$t(3) = 6.90$; $p < 0.0102$	$t(3) = 1.82$; $p < 0.1048$	–

Table S8. Statistical results based on the t-test for the determination of elongation at break for non-aged NRL gloves containing varying contents of MPP, PPP, and DPP. The values are shown as t(degrees of freedom) = t-value; p < p-value.

	**Pristine NR**	**2-phr MPP**	**4-phr MPP**	**6-phr MPP**	**2-phr PPP**	**4-phr PPP**	**6-phr PPP**	**2-phr DPP**	**4-phr DPP**	**6-phr DPP**
**Pristine NR**	–	t(3) = -1.86; p < 0.1016	t(3) = 4.33; p < 0.0247	t(3) = 3.26; p < 0.0412	t(3) = 6.30; p < 0.0121	t(3) = -5.33; p < 0.0167	t(3) = -1.90; p < 0.0986	t(3) = -4.76; p < 0.0207	t(3) = 1.94; p < 0.0962	t(3) = 20.69; p < 0.0012
**2-phr MPP**	t(3) = 1.86; p < 0.1016	–	t(3) = 2.88; p < 0.0511	t(3) = 3.09; p < 0.0454	t(3) = 3.36; p < 0.0391	t(3) = 0.16; p < 0.4443	t(3) = 0.78; p < 0.2591	t(3) = 0.29; p < 0.3981	t(3) = 1.88; p < 0.1001	t(3) = 7.57; p < 0.0085
**4-phr MPP**	t(3) = -4.33; p < 0.0247	t(3) = -2.88; p < 0.0511	–	t(3) = 2.19; p < 0.0799	t(3) = 5.65; p < 0.0150	t(3) = -16.83; p < 0.018	t(3) = -7.07; p < 0.0097	t(3) = -12.49; p < 0.0032	t(3) = -0.74; p < 0.2682	t(3) = 54.84; p < 0.0002
**6-phr MPP**	t(3) = -3.26; p < 0.0412	t(3) = -3.09; p < 0.0454	t(3) = -2.19; p < 0.0799	–	t(3) = 2.53; p < 0.0634	t(3) = -13.38; p < 0.0028	t(3) = -13.16; p < 0.0029	t(3) = -10.57; p < 0.0044	t(3) = -1.45; p < 0.1419	t(3) = 13.26; p < 0.0028
**2-phr PPP**	t(3) = -6.30; p < 0.0121	t(3) = -3.36; p < 0.0391	t(3) = -5.65; p < 0.0150	t(3) = -2.53; p < 0.0634	–	t(3) = -80.79; p < 0.0001	t(3) = -25.70; p < 0.0008	t(3) = -76.55; p < 0.0001	t(3) = -4.28; p < 0.0253	t(3) = 10.49; p < 0.0045
**4-phr PPP**	t(3) = 5.33; p < 0.0167	t(3) = -0.16; p < 0.4443	t(3) = 16.83; p < 0.018	t(3) = 13.38; p < 0.0028	t(3) = 80.79; p < 0.0001	–	t(3) = 5.25; p < 0.0172	t(3) = 3.16; p < 0.0436	t(3) = 6.60; p < 0.0111	t(3) = 29.03; p < 0.0006
**6-phr PPP**	t(3) = 1.90; p < 0.0986	t(3) = -0.78; p < 0.2591	t(3) = 7.07; p < 0.0097	t(3) = 13.16; p < 0.0029	t(3) = 25.70; p < 0.0008	t(3) = -5.25; p < 0.0172	–	t(3) = -3.65; p < 0.0337	t(3) = 3.46; p < 0.0371	t(3) = 18.04; p < 0.0015
**2-phr DPP**	t(3) = 4.76; p < 0.0207	t(3) = -0.29; p < 0.3981	t(3) = 12.49; p < 0.0032	t(3) = 10.57; p < 0.0044	t(3) = 76.55; p < 0.0001	t(3) = -3.16; p < 0.0436	t(3) = 3.65; p < 0.0337	–	t(3) = 7.10; p < 0.0096	t(3) = -23.96; p < 0.0009
**4-phr DPP**	t(3) = -1.94; p < 0.0962	t(3) = -1.88; p < 0.1001	t(3) = 0.74; p < 0.2682	t(3) = 1.45; p < 0.1419	t(3) = 4.28; p < 0.0253	t(3) = -6.60; p < 0.0111	t(3) = -3.46; p < 0.0371	t(3) = -7.10; p < 0.0096	–	t(3) = 8.70; p < 0.0065
**6-phr DPP**	t(3) = -20.69; p < 0.0012	t(3) = -7.57; p < 0.0085	t(3) = -54.84; p < 0.0002	t(3) = -13.26; p < 0.0028	t(3) = -10.49; p < 0.0045	t(3) = -29.03; p < 0.0006	t(3) = -18.04; p < 0.0015	t(3) = 23.96; p < 0.0009	t(3) = -8.70; p < 0.0065	–

Table S9. Statistical results based on the t-test for the determination of tensile strength for thermal-aged NRL gloves containing varying contents of MPP. The values are shown as t(degrees of freedom) = t-value; p < p-value.

	Pristine NR	2-phr MPP	4-phr MPP	6-phr MPP
**Pristine NR**	–	$t(3) = -15.32$; $p < 0.0021$	$t(3) = -7.54$; $p < 0.0086$	$t(3) = -4.00$; $p < 0.0285$
**2-phr MPP**	$t(3) = 15.32$; $p < 0.0021$	–	$t(3) = 1.14$; $p < 0.1856$	$t(3) = 5.16$; $p < 0.0178$
**4-phr MPP**	$t(3) = 7.54$; $p < 0.0086$	$t(3) = -1.14$; $p < 0.1856$	–	$t(3) = 10.34$; $p < 0.0046$
**6-phr MPP**	$t(3) = 4.00$; $p < 0.0285$	$t(3) = -5.16$; $p < 0.0178$	$t(3) = -10.34$; $p < 0.0046$	–

Table S10. Statistical results based on the t-test for the determination of tensile strength for gamma-aged NRL gloves containing varying contents of MPP. The values are shown as t(degrees of freedom) = t-value; p < p-value.

	Pristine NR	2-phr MPP	4-phr MPP	6-phr MPP
**Pristine NR**	–	$t(3) = -4.21$; $p < 0.0261$	$t(3) = -4.17$; $p < 0.0265$	$t(3) = -5.23$; $p < 0.0173$
**2-phr MPP**	$t(3) = 4.21$; $p < 0.0261$	–	$t(3) = 0.20$; $p < 0.4306$	$t(3) = -4.35$; $p < 0.0245$
**4-phr MPP**	$t(3) = 4.17$; $p < 0.0265$	$t(3) = -0.20$; $p < 0.4306$	–	$t(3) = -11.22$; $p < 0.0039$
**6-phr MPP**	$t(3) = 5.23$; $p < 0.0173$	$t(3) = 4.35$; $p < 0.0245$	$t(3) = 11.22$; $p < 0.0039$	–

Table S11. Statistical results based on the t-test for the determination of tensile strength for thermal-aged NRL gloves containing varying contents of PPP. The values are shown as t(degrees of freedom) = t-value; p < p-value.

	Pristine NR	2-phr PPP	4-phr PPP	6-phr PPP
**Pristine NR**	–	t(3) = -5.10; p < 0.0182	t(3) = -1.68; p < 0.1177	t(3) = 8.34; p < 0.0070
**2-phr PPP**	t(3) = 5.10; p < 0.0182	–	t(3) = 2.90; p < 0.0505	t(3) = 8.46; p < 0.0068
**4-phr PPP**	t(3) = 1.68; p < 0.1177	t(3) = -2.90; p < 0.0505	–	t(3) = 3.80; p < 0.0314
**6-phr PPP**	t(3) = -8.34 p < 0.0070	t(3) = -8.46; p < 0.0068	t(3) = -3.80; p < 0.0314	–

Table S12. Statistical results based on the t-test for the determination of tensile strength for gamma-aged NRL gloves containing varying contents of PPP. The values are shown as t(degrees of freedom) = t-value; p < p-value.

	Pristine NR	2-phr PPP	4-phr PPP	6-phr PPP
**Pristine NR**	–	t(3) = -6.31; p < 0.0121	t(3) = -1.95; p < 0.0950	t(3) = -1.89; p < 0.0999
**2-phr PPP**	t(3) = 6.31; p < 0.0121	–	t(3) = 3.69; p < 0.0332	t(3) = 5.68; p < 0.0148
**4-phr PPP**	t(3) = 1.95; p < 0.0950	t(3) = -3.69; p < 0.0332	–	t(3) = 2.15; p < 0.0820
**6-phr PPP**	t(3) = 1.89; p < 0.0999	t(3) = -5.68; p < 0.0148	t(3) = -2.15; p < 0.0820	–

Table S13. Statistical results based on the t-test for the determination of tensile strength for thermal-aged NRL gloves containing varying contents of DPP. The values are shown as t(degrees of freedom) = t-value; p < p-value.

	Pristine NR	2-phr DPP	4-phr DPP	6-phr DPP
**Pristine NR**	–	t(3) = -2.96; p < 0.0489	t(3) = -0.64; p < 0.2934	t(3) = -4.61; p < 0.0220
**2-phr DPP**	t(3) = 2.96; p < 0.0489	–	t(3) = 3.78; p < 0.0316	t(3) = 1.84; p < 0.1039
**4-phr DPP**	t(3) = 0.64; p < 0.2934	t(3) = -3.78; p < 0.0316	–	t(3) = -0.46; p < 0.3466
**6-phr DPP**	t(3) = 4.61; p < 0.0220	t(3) = -1.84; p < 0.1039	t(3) = 0.46; p < 0.3466	–

Table S14. Statistical results based on the t-test for the determination of tensile strength for gamma-aged NRL gloves containing varying contents of DPP. The values are shown as t(degrees of freedom) = t-value; p < p-value.

	Pristine NR	2-phr DPP	4-phr DPP	6-phr DPP
**Pristine NR**	–	t(3) = 0.08; p < 0.4734	t(3) = -4.86; p < 0.0199	t(3) = -1.45; p < 0.1425
**2-phr DPP**	t(3) = -0.08; p < 0.4734	–	t(3) = -22.99; p < 0.0009	t(3) = -2.51; p < 0.0643
**4-phr DPP**	t(3) = 4.86; p < 0.0199	t(3) = 22.99; p < 0.0009	–	t(3) = 4.28; p < 0.0252
**6-phr DPP**	t(3) = 1.45; p < 0.1425	t(3) = 2.51; p < 0.0643	t(3) = -4.28; p < 0.0252	–

Table S15. Statistical results based on the t-test for the determination of tensile modulus for thermal-aged NRL gloves containing varying contents of MPP. The values are shown as t(degrees of freedom) = t-value; p < p-value.

	Pristine NR	2-phr MPP	4-phr MPP	6-phr MPP
**Pristine NR**	–	t(3) = 2.93; p < 0.0497	t(3) = 5.77; p < 0.0144	t(3) = 0.89; p < 0.2343
**2-phr MPP**	t(3) = -2.93; p < 0.0497	–	t(3) = -0.05; p < 0.4823	t(3) = -2.00; p < 0.0918
**4-phr MPP**	t(3) = -5.77; p < 0.0144	t(3) = 0.05; p < 0.4823	–	t(3) = -7.57; p < 0.0085
**6-phr MPP**	t(3) = -0.89; p < 0.2343	t(3) = 2.00; p < 0.0918	t(3) = 7.57; p < 0.0085	–

Table S16. Statistical results based on the t-test for the determination of tensile modulus for gamma-aged NRL gloves containing varying contents of MPP. The values are shown as t(degrees of freedom) = t-value; p < p-value.

	Pristine NR	2-phr MPP	4-phr MPP	6-phr MPP
**Pristine NR**	–	t(3) = -3.47; p < 0.0369	t(3) = -4.82 p < 0.0202	t(3) = -1.89; p < 0.0999
**2-phr MPP**	t(3) = 3.47; p < 0.0369	–	t(3) = 2.38; p < 0.0700	t(3) = 0.54; p < 0.3206
**4-phr MPP**	t(3) = 4.82 p < 0.0202	t(3) = -2.38; p < 0.0700	–	t(3) = -0.29; p < 0.4010
**6-phr MPP**	t(3) = 1.89; p < 0.0999	t(3) = -0.54; p < 0.3206	t(3) = 0.29; p < 0.4010	–

Table S17. Statistical results based on the t-test for the determination of tensile modulus for thermal-aged NRL gloves containing varying contents of PPP. The values are shown as t(degrees of freedom) = t-value; p < p-value.

	Pristine NR	2-phr PPP	4-phr PPP	6-phr PPP
**Pristine NR**	–	t(3) = 0.92; p < 0.2278	t(3) = 3.00; p < 0.0476	t(3) = 2.85; p < 0.0522
**2-phr PPP**	t(3) = -0.92; p < 0.2278	–	t(3) = 4.21; p < 0.0260	t(3) = 4.72; p < 0.0210
**4-phr PPP**	t(3) = -3.00; p < 0.0476	t(3) = -4.21; p < 0.0260	–	t(3) = -3.14; p < 0.0441
**6-phr PPP**	t(3) = -2.85; p < 0.0522	t(3) = -4.72; p < 0.0210	t(3) = 3.14; p < 0.0441	–

Table S18. Statistical results based on the t-test for the determination of tensile modulus for gamma-aged NRL gloves containing varying contents of PPP. The values are shown as t(degrees of freedom) = t-value; p < p-value.

	Pristine NR	2-phr PPP	4-phr PPP	6-phr PPP
**Pristine NR**	–	t(3) = -3.47; p < 0.0369	t(3) = -4.82; p < 0.0202	t(3) = -1.89; p < 0.0999
**2-phr PPP**	t(3) = 3.47; p < 0.0369	–	t(3) = 2.38; p < 0.0700	t(3) = 0.54; p < 0.3206
**4-phr PPP**	t(3) = 4.82; p < 0.0202	t(3) = -2.38; p < 0.0700	–	t(3) = -0.29; p < 0.4010
**6-phr PPP**	t(3) = 1.89; p < 0.0999	t(3) = -0.54; p < 0.3206	t(3) = 0.29; p < 0.4010	–

Table S19. Statistical results based on the t-test for the determination of tensile modulus for thermal-aged NRL gloves containing varying contents of DPP. The values are shown as t(degrees of freedom) = t-value; p < p-value.

	Pristine NR	2-phr DPP	4-phr DPP	6-phr DPP
**Pristine NR**	–	t(3) = 17.67; p < 0.0016	t(3) = -3.37; p < 0.0389	t(3) = -12.74; p < 0.0031
**2-phr DPP**	t(3) = -17.67; p < 0.0016	–	t(3) = -11.96; p < 0.0035	t(3) = -18.32; p < 0.0015
**4-phr DPP**	t(3) = 3.37; p < 0.0389	t(3) = 11.96; p < 0.0035	–	t(3) = -14.38; p < 0.0024
**6-phr DPP**	t(3) = 12.74; p < 0.0031	t(3) = 18.32; p < 0.0015	t(3) = 14.38; p < 0.0024	–

Table S20. Statistical results based on the t-test for the determination of tensile modulus for gamma-aged NRL gloves containing varying contents of DPP. The values are shown as t(degrees of freedom) = t-value; p < p-value.

	Pristine NR	2-phr DPP	4-phr DPP	6-phr DPP
**Pristine NR**	–	t(3) = -12.13; p < 0.0034	t(3) = -6.18; p < 0.0126	t(3) = -46.00; p < 0.0002
**2-phr DPP**	t(3) = 12.13; p < 0.0034	–	t(3) = -3.02; p < 0.0472	t(3) = -77.48; p < 0.0001
**4-phr DPP**	t(3) = 6.18; p < 0.0126	t(3) = 3.02; p < 0.0472	–	t(3) = -5.95; p < 0.0135
**6-phr DPP**	t(3) = 46.00; p < 0.0002	t(3) = 77.48; p < 0.0001	t(3) = 5.95; p < 0.0135	–

Table S21. Statistical results based on the t-test for the determination of elongation at break for thermal-aged NRL gloves containing varying contents of MPP. The values are shown as t(degrees of freedom) = t-value; p < p-value.

	Pristine NR	2-phr MPP	4-phr MPP	6-phr MPP
**Pristine NR**	–	$t(3) = -27.01$; $p < 0.0007$	$t(3) = -26.68$; $p < 0.0007$	$t(3) = -57.11$; $p < 0.0002$
**2-phr MPP**	$t(3) = 27.01$; $p < 0.0007$	–	$t(3) = -0.15$; $p < 0.4487$	$t(3) = 7.69$; $p < 0.0082$
**4-phr MPP**	$t(3) = 26.68$; $p < 0.0007$	$t(3) = 0.15$; $p < 0.4487$	–	$t(3) = 9.62$; $p < 0.0053$
**6-phr MPP**	$t(3) = 57.11$; $p < 0.0002$	$t(3) = -7.69$; $p < 0.0082$	$t(3) = -9.62$; $p < 0.0053$	–

Table S22. Statistical results based on the t-test for the determination of elongation at break for gamma-aged NRL gloves containing varying contents of MPP. The values are shown as t(degrees of freedom) = t-value; p < p-value.

	Pristine NR	2-phr MPP	4-phr MPP	6-phr MPP
**Pristine NR**	–	$t(3) = -3.58$; $p < 0.0350$	$t(3) = -1.86$ $p < 0.1022$	$t(3) = -2.25$; $p < 0.0768$
**2-phr MPP**	$t(3) = 3.58$; $p < 0.0350$	–	$t(3) = 1.85$; $p < 0.1027$	$t(3) = 6.54$; $p < 0.0113$
**4-phr MPP**	$t(3) = 1.86$ $p < 0.1022$	$t(3) = -1.85$; $p < 0.1027$	–	$t(3) = 0.55$; $p < 0.3191$
**6-phr MPP**	$t(3) = 2.25$; $p < 0.0768$	$t(3) = -6.54$; $p < 0.0113$	$t(3) = -0.55$; $p < 0.3191$	–

Table S23. Statistical results based on the t-test for the determination of elongation at break for thermal-aged NRL gloves containing varying contents of PPP. The values are shown as t(degrees of freedom) = t-value; p < p-value.

	**Pristine NR**	**2-phr PPP**	**4-phr PPP**	**6-phr PPP**
**Pristine NR**	–	$t(3) = -5.04$; $p < 0.0186$	$t(3) = -3.44$; $p < 0.0376$	$t(3) = 1.50$; $p < 0.1360$
**2-phr PPP**	$t(3) = 5.04$; $p < 0.0186$	–	$t(3) = 0.37$; $p < 0.3749$	$t(3) = 7.76$; $p < 0.0081$
**4-phr PPP**	$t(3) = 3.44$; $p < 0.0376$	$t(3) = -0.37$; $p < 0.3749$	–	$t(3) = 5.76$; $p < 0.0144$
**6-phr PPP**	$t(3) = -1.50$; $p < 0.1360$	$t(3) = -7.76$; $p < 0.0081$	$t(3) = -5.76$; $p < 0.0144$	–

Table S24. Statistical results based on the t-test for the determination of elongation at break for gamma-aged NRL gloves containing varying contents of PPP. The values are shown as t(degrees of freedom) = t-value; p < p-value.

	**Pristine NR**	**2-phr PPP**	**4-phr PPP**	**6-phr PPP**
**Pristine NR**	–	$t(3) = -4.99$; $p < 0.0189$	$t(3) = -1.91$; $p < 0.0982$	$t(3) = -0.92$; $p < 0.2267$
**2-phr PPP**	$t(3) = 4.99$; $p < 0.0189$	–	$t(3) = 0.97$; $p < 0.2175$	$t(3) = 1.28$; $p < 0.1638$
**4-phr PPP**	$t(3) = 1.91$; $p < 0.0982$	$t(3) = -0.97$; $p < 0.2175$	–	$t(3) = 1.53$; $p < 0.1328$
**6-phr PPP**	$t(3) = 0.92$; $p < 0.2267$	$t(3) = -1.28$; $p < 0.1638$	$t(3) = -1.53$; $p < 0.1328$	–

Table S25. Statistical results based on the t-test for the determination of elongation at break for thermal-aged NRL gloves containing varying contents of DPP. The values are shown as t(degrees of freedom) = t-value; p < p-value.

	**Pristine NR**	**2-phr DPP**	**4-phr DPP**	**6-phr DPP**
**Pristine NR**	–	$t(3) = -6.03$; $p < 0.0132$	$t(3) = 0.83$; $p < 0.2478$	$t(3) = 2.48$; $p < 0.0658$
**2-phr DPP**	$t(3) = 6.03$; $p < 0.0132$	–	$t(3) = 16.68$; $p < 0.0018$	$t(3) = 4.44$; $p < 0.0235$
**4-phr DPP**	$t(3) = -0.83$; $p < 0.2478$	$t(3) = -16.68$; $p < 0.0018$	–	$t(3) = 0.99$; $p < 0.2136$
**6-phr DPP**	$t(3) = -2.48$; $p < 0.0658$	$t(3) = -4.44$; $p < 0.0235$	$t(3) = -0.99$; $p < 0.2136$	–

Table S26. Statistical results based on the t-test for the determination of elongation at break for gamma-aged NRL gloves containing varying contents of DPP. The values are shown as t(degrees of freedom) = t-value; p < p-value.

	**Pristine NR**	**2-phr DPP**	**4-phr DPP**	**6-phr DPP**
**Pristine NR**	–	$t(3) = 1.82$; $p < 0.1049$	$t(3) = -1.50$; $p < 0.1356$	$t(3) = 1.66$; $p < 0.1198$
**2-phr DPP**	$t(3) = -1.82$; $p < 0.1049$	–	$t(3) = -13.66$; $p < 0.0027$	$t(3) = 1.15$; $p < 0.1838$
**4-phr DPP**	$t(3) = 1.50$; $p < 0.1356$	$t(3) = 13.66$; $p < 0.0027$	–	$t(3) = 13.30$; $p < 0.0028$
**6-phr DPP**	$t(3) = -1.66$; $p < 0.1198$	$t(3) = -1.15$; $p < 0.1838$	$t(3) = -13.30$; $p < 0.0028$	–
